# Surgical Volume and Outcomes of Intraoperative Transesophageal Echocardiography in Coronary Artery Bypass Graft

**DOI:** 10.1001/jamanetworkopen.2025.40559

**Published:** 2025-10-30

**Authors:** Emily J. MacKay, Charlotte J. Talham, Wilson Y. Szeto, Chase R. Brown, John G. Augoustides, Nimesh D. Desai, Peter W. Groeneveld, Bo Zhang

**Affiliations:** 1 Department of Anaesthesiology and Critical Care, Perelman School of Medicine, University of Pennsylvania, Philadelphia; 2 Penn’s Cardiovascular Outcomes, Quality and Evaluative Research Center (CAVOQER), University of Pennsylvania, Philadelphia; 3 Leonard Davis Institute of Health Economics (LDI), University of Pennsylvania, Philadelphia; 4 Department of Biostatistics, University of Washington, Seattle; 5 Division of Cardiovascular Surgery, Perelman School of Medicine, University of Pennsylvania, Philadelphia; 6 Department of Internal Medicine, Perelman School of Medicine, University of Pennsylvania, Philadelphia; 7 Corporal Michael J. Crescenz Veterans Affairs Medical Center, Philadelphia, Pennsylvania; 8 Vaccine and Infectious Disease Division, Fred Hutchinson Cancer Center, Seattle, Washington

## Abstract

**Importance:**

The routine use of intraoperative transesophageal echocardiography (TEE) during coronary artery bypass graft (CABG) surgery remains controversial. Its benefit across different patient populations is unclear.

**Objectives:**

To identify patient subgroups with the greatest or least likelihood to benefit from intraoperative TEE during CABG, stratified by hospital surgical volume.

**Design, Setting, and Participants:**

This 2-stage, matched retrospective cohort study applied target trial emulation methodologies to the Society of Thoracic Surgeons (STS) Adult Cardiac Surgery Database (ACSD) to quantify the conditional treatment effect of intraoperative TEE among subpopulations undergoing isolated CABG at low, medium, and high surgical volume hospitals. The study cohort consisted of patients aged 18 years or older who underwent isolated CABG surgery between July 1, 2014, and June 30, 2022. Data analysis was conducted from August 8, 2023, to December 15, 2024.

**Exposure:**

Receipt of an intraoperative TEE during CABG surgery.

**Main Outcomes and Measures:**

The primary outcome was mortality within 30 days of surgery. Statistical analyses included multivariable logistic regression and multiple TEE vs without TEE matched comparisons stratified by surgical volume and patient subpopulations.

**Results:**

Of 1 266 055 patients who underwent isolated CABG, 963 976 (76.1%) were male, and the mean (SD) age was 65.7 (10.0) years. Among these patients, 61.8% received TEE and 39.0% did not receive TEE. Intraoperative TEE use (vs without TEE) was associated with a significant survival benefit among patients treated at hospitals with low surgical volume (2.47% vs 2.94%; odds ratio [OR], 0.83 [95% CI, 0.78-0.89], *P* < .001) and medium surgical volume (2.09% vs 2.34%; OR, 0.89 [95% CI, 0.85-0.93], *P* < .001) but not high surgical volume (1.72% vs 1.77%; OR, 0.97 [95% CI, 0.91-1.03], *P* = .48). Among patients who underwent isolated CABG at low and medium surgical volume hospitals, TEE provided the greatest survival benefit to subpopulations with greater than 50% (vs ≤50%) left-main coronary stenosis, 3 or more (vs <3) diseased coronaries, and (3) a preoperative inotropic requirement.

**Conclusions and Relevance:**

In isolated CABG, intraoperative TEE was associated with survival benefit at low- and medium-volume hospitals, particularly in patients with complex coronary disease or hemodynamic instability, but not at high-volume hospitals. These results highlight persistent equipoise and the need for randomized evaluation.

## Key Points

**Question:**

Among patients undergoing isolated coronary artery bypass graft (CABG), which subgroups derive the greatest survival benefit from intraoperative transesophageal echocardiography (TEE), and does this benefit vary by hospital surgical volume?

**Findings:**

In this cohort study of 1.26 million patients who underwent isolated CABG, intraoperative TEE was associated with survival benefits at low and medium surgical volume hospitals, particularly among patients with greater than 50% left-main coronary stenosis, 3 or more diseased vessels, or preoperative inotrope use. No survival benefit was observed at high surgical volume hospitals.

**Meaning:**

These findings support a more individualized approach to TEE use during isolated CABG surgery and provide a rationale for future randomized evaluation.

## Teaser

This cohort study examines the association of intraoperative transesophageal echocardiography use, 6 patient-level factors, and surgical volume of a hospital with survival outcomes of coronary artery bypass graft (CABG).

## Introduction

Transesophageal echocardiography (TEE) is used during cardiac surgery to guide intraoperative decisions and is associated with improved outcomes after valve and aortic procedures.^1,2,3^ However, its benefit in isolated coronary artery bypass graft (CABG) remains uncertain, particularly for low-risk patients. While retrospective studies suggest TEE may improve outcomes in sicker patients who undergo CABG,^4,5,6^ the overall treatment effect is marginal^5,7^ and negligible in healthier subgroups.^5,7^

Beyond clinical equipoise, hospital-level resource limitations and staffing shortages affect TEE use during CABG.^8,9^ At TEE resource–limited hospitals, a precision medicine refinement for TEE allocation could improve outcomes by targeting patients most likely to benefit from TEE.

To advance prior observational work,^4,5,6,7^ this study aimed to identify patient subgroups with the greatest or least likelihood to benefit from intraoperative TEE during CABG, stratified by hospital surgical volume. We hypothesized that the association between TEE use and survival would vary by patient characteristics and hospital surgical volume. To accomplish this goal rigorously, we leveraged data from the Society of Thoracic Surgeons (STS) Adult Cardiac Surgery Database (ACSD) in conducting a 2-stage analysis with a target trial emulation framework^10,11,12,13^ to uncover heterogeneity in TEE’s survival benefit across subgroups.

## Methods

### Data Source and Study Population

The STS ACSD contains more than 8 million surgical records from more than 95% of US cardiac surgical centers.^14^ We queried data from versions 2.81, 2.90, and 4.20.2 of the database. All data management and statistical analyses were performed in accordance with the STS National Database Participant User Files Research Program data use agreement. We followed the Strengthening the Reporting of Observational Studies in Epidemiology (STROBE) reporting guideline for cohort studies.^15^ The Advarra Institutional Review Board deemed this study exempt from review and waived the informed consent requirement because deidentified data were used.

The study cohort included all adults (aged ≥18 years) who underwent isolated CABG from July 1, 2014, to June 30, 2022. Exclusions were missing TEE data, concomitant non-CABG cardiac surgery (eg, valve, structural, and aortic), missing hospital indicator, missing diseased vessel count, and emergent salvage or unknown operative status. We elected to exclude observations with missing data in these covariates because of the size of our dataset.

### Exposure and Outcome

The exposure was receipt of an intraoperative TEE during CABG surgery. Used in prior studies,^3,5,9^ intraoperative TEE is consistent across all versions of the STS ACSD used in this analysis.

The outcome was operative mortality. Operative mortality was defined as meeting either one of the following criteria: all-cause death occurring during the index hospitalization (including >30 days) or all-cause death after discharge but fewer than 30 days after the index surgery.^16^

### Covariates for Matching

A comprehensive collection of independent covariates was used for matching to embed observational data into a target randomized trial (eAppendix 1 in Supplement 1). These covariates included demographics (age, sex, and race), admission status, year of surgery, preoperative comorbidities, hemodynamic parameters, laboratory values, STS Predicted Risk of Mortality (PROM) score, and hospital surgical volume (eAppendix 2 in Supplement 1). Race (categorized as American Indian or Alaska Native, Asian, Black, White, and other [including all responses other than the categories shown]) was included according to STS ACSD definitions (eFigure 1 and eTable 1 in Supplement 1).

All variables used in this analysis are standardized in the STS ACSD and are intermittently validated at individual centers by the STS Research and Analytic Center. Missing data rates among all variables included in the analyses did not exceed 0.001%, and single imputation (using median for continuous variables and mode for categorical variables) was used to address the minor residual missingness for included variables.

### Potential Covariates Modifying the Treatment Effect of TEE 

Six clinically relevant covariates were included in the logistic regression to estimate the conditional average treatment effect (CATE) of TEE in isolated CABG: (1) ejection fraction, (2) congestive heart failure (CHF; defined as having a New York Heart Association class I-IV^16^), (3) preoperative serum creatinine, (4) greater than 50% left-main coronary stenosis, (5) 3 or more diseased coronaries, and (6) inotrope use within 48 hours of surgery. These variables were selected based on a combination of clinical judgment, prior evidence of effect modification,^6^ prior utilization, and electronic medical record data availability.

### Surgical Volume

Because CABG survival improves at higher surgical volume hospitals,^17,18^ we stratified analyses by surgical volume to reduce hospital-level confounding between TEE use and survival. Based on prior literature, surgical volume was classified as low with fewer than 100, as medium with 100 to 250, and as high with more than 250 annual isolated CABG cases using established volume thresholds.^17,18,19,20,21^

### Study Design

**Figure 1.**
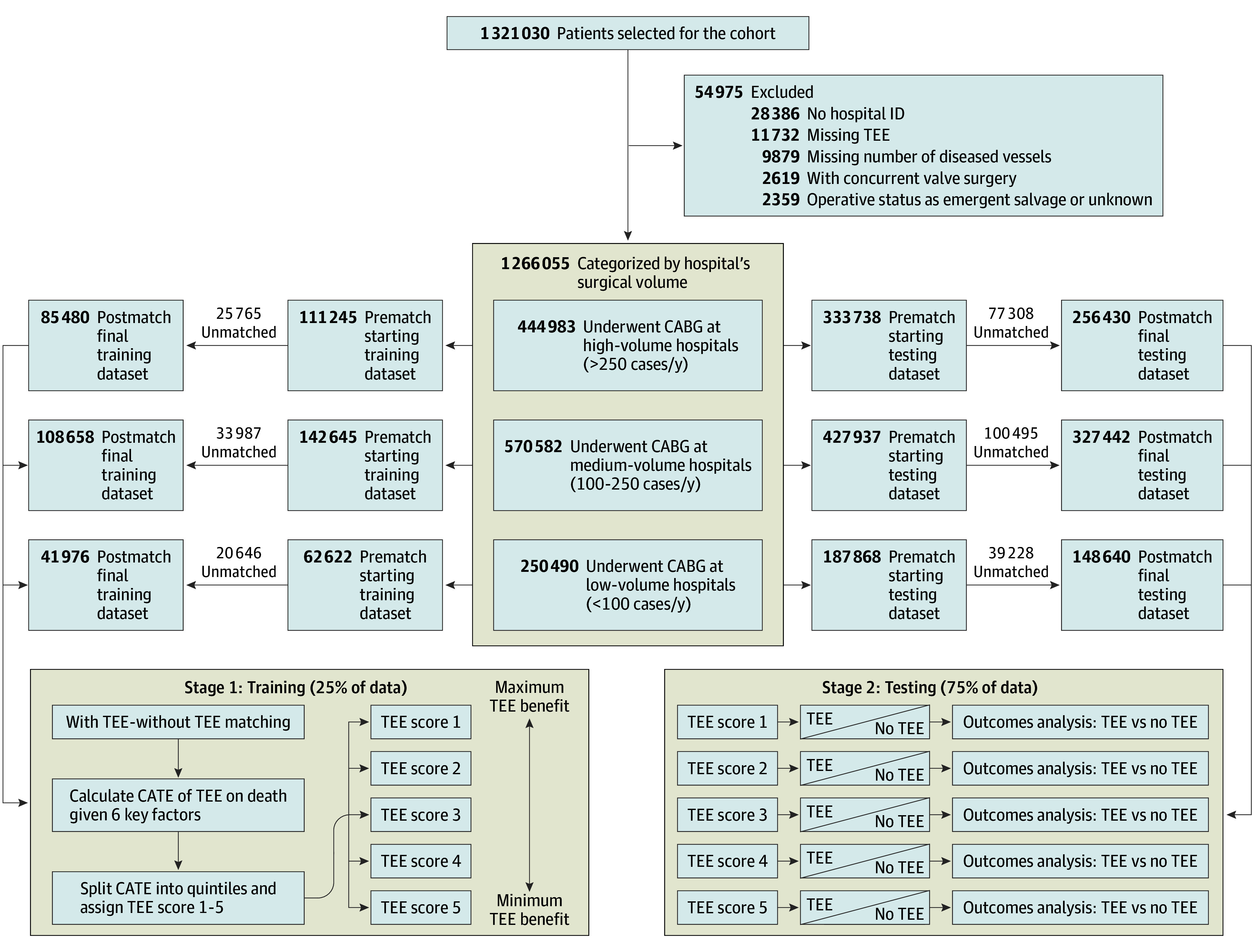
Study Flow Diagram Stage 1 shows derivation of the transesophageal echocardiography (TEE) score, calculated as the conditional average treatment effect (CATE) of intraoperative TEE on mortality based on 6 factors (ejection fraction, congestive heart failure, preoperative serum creatinine, >50% left-main coronary stenosis, ≥3 diseased coronaries, and preoperative inotrope use). Stage 2 shows validation of the TEE score (range: 1-5, with 1 indicating most benefit and 5 indicating least benefit from TEE) via matched cohort analysis within each surgical volume stratum and score quintile. CABG indicates coronary artery bypass graft; ID, identification.

Using target trial emulation methodologies,^10,11,12,13^ consistent with prior work,^13,22,23^ we conducted a 2-stage analysis to estimate the CATE of intraoperative TEE in isolated CABG. In stage 1, we identified subgroups who may derive the greatest and least benefit from TEE based on the 6 preselected covariates (eAppendix 4 in Supplement 1). In stage 2, we used matched cohort studies to compare operative mortality with TEE vs without TEE among the 5 TEE score categories derived in stage 1 (Figure 1; eAppendix 5 in Supplement 1).

#### Stage 1: Identification of Subpopulations Who Benefit From TEE, and Derivation of the TEE Score 

Estimating the CATE for intraoperative TEE use during isolated CABG surgery involved several steps. First, within each surgical volume strata (low, medium, and high), on a random 25% subset of data, we emulated randomization using statistical matching^24^ to ensure baseline covariate balance among patients who underwent isolated CABG with TEE vs without TEE. Second, we used logistic regression to model operative mortality with TEE vs without TEE conditional on the 6 key covariates (ejection fraction, CHF, preoperative serum creatinine, >50% left-main coronary stenosis, ≥3 diseased coronaries, and preoperative inotrope use). The CATE for intraoperative TEE was calculated as the difference in probability of operative mortality with TEE minus without TEE. The cohort was then divided into equal quintiles and assigned a numeric value—termed TEE score—ranging from 1 (indicating greatest TEE benefit) to 5 (indicating least TEE benefit).

#### Stage 2: Validation of the TEE Score via Multiple Matched Comparisons 

To test whether the 5 TEE score categories would correctly estimate the clinical benefit of TEE (vs without TEE) after isolated CABG, we conducted a series of matched cohort studies using the reserved 75% subset of data. Within each surgical volume category (low, medium, and high), we compared survival with TEE vs without TEE for each TEE score subgroup.

### Statistical Matching

To ensure baseline covariate balance between patients who underwent isolated CABG with TEE vs without TEE, 1:1 pair matching was performed. Consistent with prior comparative effectiveness studies,^3,6,25,26^ both stages 1 and 2 used exact matching^27^ and fine balancing^28^ on selected variables, along with propensity score–balanced matching for all remaining covariates.^24^ Statistical matching used the Mahalanobis distance with a propensity score caliper and balanced the estimated propensity score distribution, as implemented using the match2C package^24^ in R (R Project for Statistical Computing). Details are provided in eAppendix 3 in Supplement 1.

### Statistical Analysis

Matching quality in both the training (stage 1) and testing (stage 2) phases was assessed using standardized mean differences (SMDs), with SMD less than 0.10 indicating acceptable balance.^29,30^ Baseline covariate distributions overall and by TEE score were summarized using descriptive statistics. Operative mortality was analyzed using the McNemar test.^31^ Within each TEE score subgroup, both the risk difference (RD) and odds ratio (OR) were reported.

To assess the robustness of our findings, we conducted 2 sensitivity analyses. First, we calculated E-values^32^ to quantify the minimum strength of association that an unmeasured confounder would need to have with both TEE use and the outcome in order to fully explain the observed association. Second, we conducted a negative control outcome analysis using new-onset postoperative atrial fibrillation (an event not plausibly affected by intraoperative TEE) to evaluate whether residual unmeasured confounding was adequately controlled.

In supplemental analyses, we also compared rates of coronary reintervention, postoperative stroke, and postoperative chest exploration for bleeding between the with TEE and without TEE groups. All hypothesis testing was 2-sided, and statistical significance was set at *P* < .05. Data preprocessing was performed in Stata, version 18.0 (StataCorp LLC). Matching and statistical analyses were conducted using the dplyr, exact2x2, and match2C^33^ packages in R, version 4.2.2 (R Project for Statistical Computing). Code is available in a GitHub repository (link provided in eAppendix 13 in Supplement 1). Data analysis was conducted from August 8, 2023, to December 15, 2024.

## Results

Following exclusions (Figure 1), our study cohort consisted of 1 266 055 patients who underwent isolated CABG surgery. Of these patients, 963 976 (76.1%) were male and 302 079 (23.9%) were female, with a mean (SD) age of 65.7 (10.0) years, and 0.7% identified as American Indian or Alaska Native, 3.6% as Asian, 7.5% as Black, 84.3% as White, and 4.1% as individuals of other race.

Among the 1 255 055 isolated CABG procedures, 61.8% received TEE and 39.0% did not. Patients with TEE were demographically and hemodynamically similar to patients without TEE, including mean (SD) ejection fraction (51.87% [12.35%] vs 52.98% [11.61%]) and pulmonary artery systolic pressure (30.89 [6.46] vs 30.95 [6.99] mm Hg) laboratory values, prior procedures (both 1.6%), and mean (SD) PROM score (both 0.02% [0.03%]; lower score indicating lower risk and higher score indicating higher risk of mortality) (Table 1). Distribution across hospitals by surgical volume was comparable across low (19.8%), medium (45.1%), and high (35.1%) volumes (Table 1). Patients without TEE compared with patients with TEE were more often elective cases (39.5% vs 36.8%), were less often transfer admissions (22.7% vs 25.8%), and had lower CHF rates (17.7% vs 21.1%) (Table 1).

**Table 1.** Unadjusted Baseline Characteristics of the Isolated Coronary Artery Bypass Graft Cohort

Covariate^a^	Patients, No. (%)		
	Overall	Without TEE	With TEE
No.	1 266 055	489 895	776 160
Age, mean (SD), y	65.7 (10.0)	65.7 (10.1)	65.6 (10.0)
Sex			
Female	302 079 (23.9)	119 864 (24.5)	182 215 (23.5)
Male	963 976 (76.1)	370 031 (75.5)	593 945 (76.5)
Race^b^			
American Indian or Alaska Native	8484 (0.7)	3547 (0.7)	4937 (0.6)
Asian	45 668 (3.6)	14 140 (2.9)	31 528 (4.1)
Black	94 324 (7.5)	36 395 (7.4)	57 929 (7.5)
White	1 067 823 (84.3)	419 472 (85.6)	648 351 (83.5)
Other^c^	52 312 (4.1)	17 397 (3.6)	34 915 (4.5)
Admit source			
Elective	591 748 (46.7)	237 428 (48.5)	354 320 (45.7)
Emergency	335 980 (26.5)	131 432 (26.8)	204 548 (26.4)
Transfer	311 583 (24.6)	111 275 (22.7)	200 308 (25.8)
Arrhythmia	185 899 (14.7)	67 462 (13.8)	118 437 (15.3)
CHF	250 227 (19.8)	86 571 (17.7)	163 706 (21.1)
CVD	278 608 (22.0)	104 953 (21.4)	173 655 (22.4)
Laboratory values			
PA systolic pressure, mean (SD), mm Hg	30.93 (6.79)	30.89 (6.46)	30.95 (6.99)
Ejection fraction, mean (SD)	52.30 (12.08)	52.98 (11.61)	51.87 (12.35)
Previous CABG	20 192 (1.6)	7875 (1.6)	12 317 (1.6)
Hemoglobin, mean (SD), g/dL	13.25 (1.98)	13.28 (1.97)	13.23 (1.99)
INR, mean (SD)	1.05 (0.24)	1.05 (0.24)	1.06 (0.23)
Creatinine, mean (SD), mg/dL	1.20 (1.12)	1.18 (1.05)	1.22 (1.16)
>50% Left-main coronary stenosis	405 296 (32.0)	154 524 (31.5)	250 772 (32.3)
≥3 Diseased coronaries	974 761 (77.0)	371 480 (75.8)	603 281 (77.7)
Inotrope use within 48 h of surgery	14 673 (1.2)	5238 (1.1)	9435 (1.2)
Operative status			
Elective	478 614 (37.8)	193 351 (39.5)	285 263 (36.8)
Emergent	46 519 (3.7)	18 094 (3.7)	28 425 (3.7)
Urgent	740 922 (58.5)	278 450 (56.8)	462 472 (59.6)
STS PROM score, mean (SD), %^d^	0.02 (0.03)	0.02 (0.03)	0.02 (0.03)
Hospital surgical volume			
Low	250 490 (19.8)	99 179 (20.2)	151 311 (19.5)
Medium	570 582 (45.1)	218 053 (44.5)	352 529 (45.4)
High	444 983 (35.1)	172 663 (35.2)	272 320 (35.1)

Abbreviations: CABG, coronary artery bypass graft; CHF, congestive heart failure; CVD, cerebrovascular disease; INR, international normalized ratio; PA, pulmonary artery; PROM, Predicted Risk of Mortality; STS, Society of Thoracic Surgeons; TEE, transesophageal echocardiography.

SI conversion factors: To convert creatinine to micromoles per liter, multiply by 88.4; hemoglobin level to gram per liter, multiply by 10.0.

^a^ Covariates represent a selection. The full, baseline covariate distributions may be viewed in eTable 1 in Supplement 1.

^b^ Race data were obtained from STS Adult Cardiac Surgery Database.

^c^ Other included all responses other than the categories shown.

^d^ PROM: lower score indicating lower risk and higher score indicating higher risk of mortality.

### Matching Assessment

In stage 1, prior TEE score development postmatch SMDs for all 40 covariates across the 3 surgical volume groups were less than 0.01, well below the accepted SMD threshold (<0.10).^29,30^ Covariate balance for the low, medium, and high surgical volume hospitals is shown in eAppendix 7 and eTables 3 and 4; eAppendix 8 and eTables 5 and 6; and eAppendix 9 and eTables 7 and 8 in Supplement 1. In stage 2, each of the 5 TEE score categories also achieved SMD less than 0.01 for all covariates.

### Heterogeneity of Intraoperative TEE Effect by Surgical Volume

Using the postmatch training data, TEE was associated with a significant survival benefit at low surgical volume hospitals (RD, −0.419% [95% CI, −0.742% to −0.097%], *P* = .01; OR, 0.86 [95% CI, 0.77-0.97], *P* = .01) and a statistically significant but marginally clinically significant survival benefit at medium surgical volume hospitals (RD, −0.236% [95% CI, −0.410% to −0.061%], *P* = .008; OR, 0.90 [95% CI, 0.83-0.97], *P* = .009). No survival benefit was observed with TEE use at high surgical volume hospitals (RD, −0.112% [95% CI, −0.284% to 0.059%], *P* = .20; OR, 0.93 [95% CI, 0.84-1.04], *P* = .21) (eAppendix 6 and eTable 2 in Supplement 1). These findings support developing separate TEE scores by surgical volume category.

Using the reserved postmatch testing data, these findings were confirmed. TEE (vs without TEE) was associated with a significant survival benefit at low surgical volume hospitals (2.47% vs 2.94%; RD, −0.470% [95% CI, −0.631% to −0.308%], *P* < .001; OR, 0.83 [95% CI, 0.78-0.89], *P* < .001). TEE was associated with a statistically significant but marginally clinically significant survival benefit at medium surgical volume hospitals (2.09% vs 2.34%; RD, −0.245% [95% CI, −0.344% to −0.146%], *P* < .001; OR, 0.89 [95% CI, 0.85-0.93], *P* < .001). No survival benefit was observed with TEE use at high surgical volume hospitals (1.72% vs 1.77%; RD, −0.00% [95% CI, −0.0001% to 0.0001%], *P* = .45; OR, 0.97 [95% CI, 0.91-1.03], *P* = .48) (Table 2).

**Table 2.** Individualized Treatment Effects of Transesophageal Echocardiography in Coronary Artery Bypass Graft Procedures at Low, Medium, and High Surgical Volume Hospitals

TEE score^a^	TEE group	No. of patients	Death		RD (95% CI), %	*P* value^b^	OR (95% CI)	*P* value^b^
			No.	Mean rate (SE), %				
**Low surgical volume**								
Pooled	Without	74 320	2183	2.94 (0.06)	−0.470 (−0.631 to −0.308)	<.001	1 [Reference]	<.001
Pooled	With	74 320	1834	2.47 (0.06)			0.83 (0.78 to 0.89)	
1	Without	13 695	691	5.05 (0.19)	−0.701 (−1.191 to −0.211)	.009	1 [Reference]	.01
1	With	13 695	595	4.34 (0.17)			0.85 (0.76 to 0.95)	
2	Without	14 217	391	2.75 (0.14)	−0.591 (−0.945 to −0.236)	.003	1 [Reference]	.003
2	With	14 217	307	2.16 (0.12)			0.78 (0.66 to 0.94)	
3	Without	15 541	316	2.05 (0.11)	−0.418 (−0.715 to −0.121)	.009	1 [Reference]	.01
3	With	15 541	253	1.63 (0.10)			0.79 (0.67 to 0.94	
4	Without	16 477	277	1.68 (0.10)	−0.334 (−0.595 to −0.072)	.01	1 [Reference]	.02
4	With	16 477	222	1.35 (0.09)			0.80 (0.66 to 0.94)	
5	Without	14 390	506	3.52 (0.15)	−0.314 (−0.745 to 0.064)	.10	1 [Reference]	.11
5	With	14 390	457	3.18 (0.15)			0.90 (0.78 to 1.02)	
**Medium surgical volume**								
Pooled	Without	163 721	3827	2.34 (0.04)	−0.245 (−0.344 to −0.146)	<.001	1 [Reference]	<.001
Pooled	With	163 721	3426	2.09 (0.04)			0.89 (0.85 to 0.93)	
1	Without	32 633	1093	3.35 (0.10)	−0.472 (−0.732 to −0.212)	<.001	1 [Reference]	.001
1	With	32 633	939	2.88 (0.09)			0.85 (0.77 to 0.93)	
2	Without	32 842	804	2.45 (0.09)	−0.298 (−0.525 to −0.072)	.02	1 [Reference]	.02
2	With	32 842	706	2.15 (0.08)			0.87 (0.79 to 0.97)	
3	Without	32 099	441	1.37 (0.07)	−0.206 (−0.377 to −0.034)	.03	1 [Reference]	.03
3	With	32 099	375	1.17 (0.06)			0.85 (0.73 to 0.98)	
4	Without	33 287	709	2.13 (0.08)	−0.117 (−0.329 to 0.095)	.28	1 [Reference]	.29
4	With	33 287	670	2.01 (0.08)			0.94 (0.84 to 1.05)	
5	Without	32 860	780	2.37 (0.08)	−0.134 (−0.358 to 0.091)	.28	1 [Reference]	.29
5	With	32 860	736	2.24 (0.08)			0.94 (0.85 to 1.04)	
**High surgical volume**								
Pooled	Without	128 215	2265	1.77 (0.04)	0 (−0.0001 to 0.0001)	.45	1 [Reference]	.48
Pooled	With	128 215	2203	1.72 (0.04)			0.97 (0.91 to 1.03)	
1	Without	25 296	714	2.82 (0.10)	0.229 (−0.058 to 0.516)	.35	1 [Reference]	.36
1	With	25 296	772	3.05 (0.11)			1.09 (0.98 to 1.21)	
2	Without	26 372	300	1.14 (0.07)	−0.053 (−0.229 to 0.123)	.56	1 [Reference]	.58
2	With	26 372	286	1.08 (0.06)			0.95 (0.90 to 1.13)	
3	Without	25 049	326	1.30 (0.07)	−0.088 (−0.282 to 0.107)	.45	1 [Reference]	.48
3	With	25 049	304	1.21 (0.07)			0.93 (0.79 to 1.09)	
4	Without	25 473	349	1.37 (0.07)	−0.185 (−0.379 to −0.010)	.35	1 [Reference]	.36
4	With	25 473	302	1.19 (0.07)			0.86 (0.74 to 1.01)	
5	Without	26 025	576	2.21 (0.09)	−0.142 (−0.387 to 0.102)	.45	1 [Reference]	.48
5	With	26 025	539	2.07 (0.09)			0.93 (0.82 to 1.05)	

Abbreviations: OR, odds ratio; RD, risk difference; TEE, transesophageal echocardiography.

^a^ TEE score range: 1 to 5, with 1 indicating most benefit and 5 indicating least benefit from TEE.

^b^ Benjamini-Hochberg correction calculation was applied to *P* values for both the RDs and ORs to control for a false discovery rate.

### Low Surgical Volume Hospitals: Individualized TEE Treatment Effects

**Figure 2.**
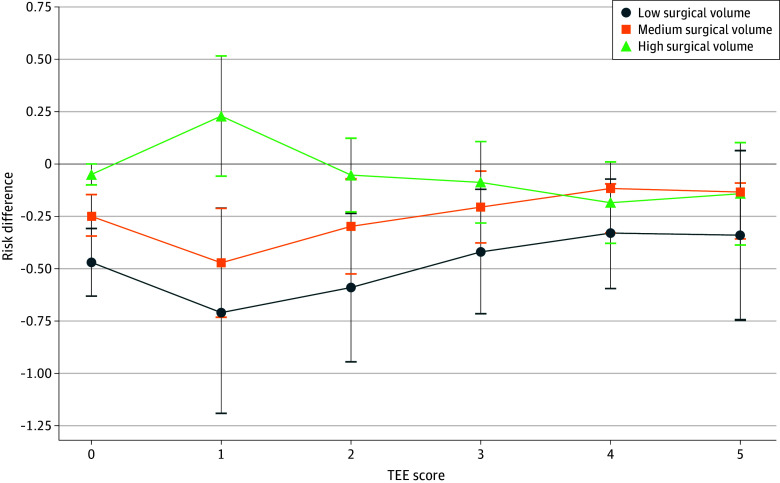
Risk Difference for Death With vs Without Transesophageal Echocardiography (TEE) in Coronary Artery Bypass Graft (CABG) by Surgical Volume and TEE Score Error bars represent 95% CIs. TEE score range: 1 to 5, with 1 indicating most benefit and 5 indicating least benefit from TEE.

Using the postmatch testing data, among 148 640 patients who underwent CABG (74 320 with TEE vs 74 320 without TEE) at low surgical volume hospitals, TEE was associated with a statistically and clinically significant survival benefit for TEE scores 1 to 3 (RD, −0.701% [95% CI, −1.191% to −0.211%] to −0.418% [95% CI, −0.715% to −0.121%]; OR, 0.78 [95% CI, 0.66-0.94] to 0.85 [95% CI, 0.76-0.95]; *P* < .01) (Table 2). TEE score 4 showed a statistically significant but marginal clinical benefit (RD, −0.334% [95% CI, −0.595 to −0.072]; OR, 0.80 [95% CI, 0.66-0.94]; *P* ≤ .01). No statistically significant benefit was observed for TEE score 5 (RD, −0.314% [95% CI, −0.745% to 0.064%]; OR, 0.90 [95% CI, 0.78-1.02]; *P* ≥ .10) (Table 2, Figure 2).

### Low Surgical Volume Hospitals: Patient Characteristics Associated With Estimated Individualized TEE Benefit

At low surgical volume hospitals vs overall, patients who benefitted most from TEE (score 1) had a lower (<55%) ejection fraction (86.8% vs 41.5%), higher creatinine level (2.02 vs 1.18 mg/dL), greater than 50% left-main stenosis (50.8% vs 33.1%), 3 or more diseased coronaries (95.6% vs 76.9%), and greater preoperative inotrope use (4.7% vs 1.2%) (Table 3; eAppendix 10 and eTable 9 in Supplement 1). Patients who benefitted least from TEE (score 5) had higher CHF prevalence (73.0% vs 15.1%) compared with the overall population (Table 3; eTable 9 in Supplement 1). Notably, patients in both TEE scores 1 and 5 groups had similarly elevated PROM score (>2.5%), compared with lower-risk patients with TEE scores 2 to 3 (<1.6%), underscoring that TEE benefit varied independent of baseline surgical risk (Table 3; eTable 9 and eAppendix 14 in Supplement 1).

**Table 3.** Characteristics Associated With Individualized Treatment Effects of Transesophageal Echocardiography at Low and Medium Surgical Volume Hospitals

Characteristic	Overall, No. (%)		TEE score, No. (%)^a^			
			1		5	
	Without TEE	With TEE	Without TEE	With TEE	Without TEE	With TEE
**Low surgical volume**						
No. of patients	74 320	74 320	13 695	13 695	14 390	14 390
EF, mean (SD)	52.54 (11.82)	52.50 (11.91)	44.73 (10.69)	44.65 (10.62)	49.17 (14.39)	48.97 (14.53)
EF <55%	30 732 (41.4)	30 879 (41.5)	11 770 (85.9)	11 886 (86.8)	6737 (46.8)	6846 (47.6)
CHF	11 312 (15.2)	11 211 (15.1)	521 (3.8)	487 (3.6)	10 519 (73.1)	10 508 (73.0)
Creatinine, mean (SD), mg/dL	1.19 (1.09)	1.18 (1.05)	2.07 (2.23)	2.02 (2.15)	1.02 (0.52)	1.02 (0.53)
>50% Left-main coronary stenosis	24 574 (33.1)	24 611 (33.1)	6912 (50.5)	6961 (50.8)	3818 (26.5)	3855 (26.8)
≥3 Diseased coronaries	56 751 (76.4)	57 135 (76.9)	13 088 (95.6)	13 090 (95.6)	8192 (56.9)	8259 (57.4)
Inotrope use within 48 h of surgery	925 (1.2)	893 (1.2)	671 (4.9)	650 (4.7)	241 (1.7)	237 (1.6)
STS PROM score, mean (SD), %^b^	1.85 (2.91)	1.84 (2.88)	3.00 (4.19)	2.95 (4.06)	2.42 (3.92)	2.44 (3.99)
Death rate, mean (SE), %	2.94 (0.06)	2.47 (0.06)	5.05 (0.19)	4.34 (0.17)	3.52 (0.15)	3.18 (0.15)
E-value	1.51	1.51	1.28	1.28	1.00	1.00
**Medium surgical volume**						
No. of patients	163 721	163 721	32 633	32 633	32 860	32 860
EF, mean (SD)	53.00 (11.68)	52.99 (11.73)	53.15 (11.35)	53.17 (11.42)	47.34 (12.82)	47.35 (12.96)
EF <55%	64 822 (39.6)	65 426 (40.0)	13 071 (40.1)	13 319 (40.8)	21 422 (65.2)	21 425 (65.2)
CHF	28 540 (17.4)	28 324 (17.3)	696 (2.1)	657 (2.0)	11 419 (34.8)	11 352 (34.5)
Creatinine, mean (SD), mg/dL	1.17 (1.02)	1.16 (0.97)	1.30 (1.32)	1.28 (1.29)	1.32 (1.34)	1.29 (1.24)
>50% Left-main coronary stenosis	51 914 (31.7)	51 737 (31.6)	31 149 (95.5)	31 289 (95.9)	2531 (7.7)	2386 (7.3)
≥3 Diseased coronaries	124 996 (76.3)	125 551 (76.7)	32 215 (98.7)	32 322 (99.0)	9086 (27.7)	9176 (27.9)
Inotrope use within 48 h of surgery	1804 (1.1)	1645 (1.0)	1804 (5.5)	1645 (5.0)	0	0
STS PROM score, mean (SD), %^b^	1.83 (3.02)	1.83 (2.98)	2.42 (4.32)	2.41 (4.31)	1.96 (3.04)	1.95 (3.02)
Death rate, mean (SE), %	2.34 (0.04)	2.09 (0.04)	3.35 (0.10)	2.88 (0.09)	2.37 (0.08)	2.24 (0.08)
E-value	1.35	1.35	1.36	1.36	1.00	1.00

Abbreviations: CHF, congestive heart failure; EF, ejection fraction; PROM, Predicted Risk of Mortality; STS, Society of Thoracic Surgeons; TEE, transesophageal echocardiography.

SI conversion factgor: To convert creatinine to µmol/L, multiply by 88.4.

^a^ TEE score range: 1 to 5, with 1 indicating most benefit and 5 indicating least benefit from TEE. The complete table with results from all 5 TEE score subgroups is available in eTable 9 (low volume) and eTable 10 (medium volume) in Supplement 1.

^b^ PROM: a lower score indicates lower risk and a higher score indicates higher risk of mortality.

### Medium Surgical Volume Hospitals: Individualized TEE Treatment Effects

Using the postmatch testing data, among 347 442 patients who underwent CABG (163 721 with TEE vs 163 721 without TEE) at medium surgical volume hospitals, TEE score 1 was associated with a statistically and clinically significant survival benefit (RD, −0.472% [95% CI, −0.732% to −0.212%]; OR, 0.85 [95% CI, 0.77-0.93]; *P* < .001). Among those with TEE scores 2 and 3, we observed a statistically significant but marginal clinical benefit (RD, −0.298% [95% CI, −0.525% to −0.072%] and −0.206% [95% CI, −0.377% to −0.034%]; OR, 0.87 [95% CI, 0.79-0.97] and 0.85 [95% CI, 0.73-0.98]; *P* = .03). No statistically significant benefit was observed for TEE scores 4 or 5 (RD, −0.117% [95% CI, −0.329% to 0.095%] and −0.134% [95% CI, −0.358% to 0.091%]; OR, 0.94 [95% CI, 0.84-1.05] and 0.94 [95% CI, 0.85-1.04]; *P* > .29) (Table 2, Figure 2).

### Medium Surgical Volume Hospitals: Patient Characteristics Associated With Estimated Individualized TEE Benefit

At medium surgical volume hospitals vs overall, patients who benefitted most from TEE (score 1) had more (>50%) left-main stenosis (95.9% vs 31.6%), 3 or more diseased coronaries (99.0% vs 76.7%), and higher preoperative inotrope use (5.0% vs 1.0%) (Table 3; eTable 10 in Supplement 1). All TEE score subgroups had similar PROM score (1.1% to 2.4%), reinforcing that TEE benefit varied independent of baseline risk (Table 3; eTable 10 and eAppendix 14 in Supplement 1).

### High Surgical Volume Hospitals: Individualized TEE Treatment Effects

Using the postmatch testing data, among 256 430 patients who underwent CABG (128 215 with TEE vs 128 215 without TEE) at high surgical volume hospitals, TEE was not associated with a statistically significant survival benefit overall or within any TEE score subgroup (Table 2, Figure 2).

### Sensitivity and Negative Control Outcome Analyses

The sensitivity analysis showed that the primary finding—intraoperative TEE use was associated with lower mortality rates in low and medium surgical volume hospitals—was robust to a moderate level of unmeasured confounding (E-value = 1.51 for low surgical volume hospitals, and 1.35 for medium surgical volume hospitals) (Table 3). Within each surgical volume strata, the association was most robust in the TEE score 1 subgroup (Table 3).

Postoperative atrial fibrillation is an outcome that should not plausibly be associated with intraoperative TEE. A negative control outcome analysis showed that, across all hospital volume strata, TEE use was associated with slightly higher rates of postoperative atrial fibrillation in pooled analyses. The pooled ORs were as follows: 1.04 (95% CI, 1.01-1.06) for low surgical volume hospitals, 1.05 (95% CI, 1.03-1.07) for medium surgical volume hospitals, and 1.06 (95% CI, 1.04-1.08) for high surgical volume hospitals. Absolute risk differences were small (<1.1%) and not consistent across TEE score subgroups (eAppendix 11 in Supplement 1). Additionally, these results are opposite to the increased survival association observed with TEE in the low and medium surgical volume hospitals from the primary analysis. This opposite-direction association for a negative control outcome strengthens the interpretation that the observed mortality benefit associated with TEE is unlikely to be explained by a systematic bias favoring TEE.

### Supplemental Clinical Outcomes

We also examined several other postoperative complications as supplemental clinical outcomes (eAppendix 11 in Supplement 1). These included coronary reintervention, postoperative stroke, and postoperative chest exploration for bleeding. These are complications for which a causal association with TEE is at least plausible. Across all hospital volume strata, we found no statistically significant differences in the rates of these outcomes between the with TEE and without TEE groups.

For coronary reintervention, pooled ORs were as follows: 1.13 (95% CI, 0.96-1.34) for low surgical volume hospitals, 0.98 (95% CI, 0.87-1.09) for medium surgical volume hospitals, and 1.12 (95% CI, 0.97-1.29) for high surgical volume hospitals. For new postoperative stroke, the pooled ORs were as follows: 1.00 (95% CI, 0.92-1.09) for low surgical volume hospitals, 1.02 (95% CI, 0.96-1.09) for medium surgical volume hospitals, and 1.00 (95% CI, 0.94-1.08) for high surgical volume hospitals. For postoperative chest exploration, the pooled ORs were as follows: 1.00 (95% CI, 0.92-1.08) for low surgical volume hospitals, 0.95 (95% CI, 0.90-1.00) for medium surgical volume hospitals, and 1.04 (95% CI, 0.97-1.10) for high surgical volume hospitals. Absolute risk differences for all 3 outcomes were small (≤0.41% for coronary reintervention, ≤1.38% for stroke, and ≤1.87% for chest exploration) and varied in direction by TEE score subgroup. These null findings may be attributable, at least in part, to the low incidence of these complications in our study population, which limited statistical power to detect small differences.

## Discussion

In this cohort study of 1 266 055 patients who underwent isolated CABG with or without intraoperative TEE, we used target trial methodologies to develop and validate individualized TEE treatment decision rules. Two key findings emerged. First, of patients undergoing isolated CABG at low (<100 cases/y) and medium (100-250 cases/y) surgical volume hospitals, our 2-stage analysis uncovered an association between intraoperative TEE and improved survival among patients (1) with greater than 50% (vs ≤50%) left-main stenosis, (2) with 3 or more (vs <3) diseased coronaries, and (3) taking inotropes (vs no inotropes) within 48 hours of surgery. Second, in patients who underwent isolated CABG at high (≥250 cases/y) surgical volume hospitals, we observed no association between intraoperative TEE (vs without TEE) and increased survival, either overall or among any subpopulations.

Current American College of Cardiology/American Heart Association guidelines assign intraoperative TEE a class IIb recommendation for isolated CABG, citing unknown usefulness.^34^ In the absence of randomized clinical trial evidence, this classification reflects uncertainty about the clinical benefit of TEE during isolated CABG surgery.^5,6,7^ Our findings contribute to a growing body of work^4,5,6,7^ that compares clinical outcomes after isolated CABG with vs without intraoperative TEE. At low and medium surgical volume centers, the results broadly align with previous observational studies showing that TEE benefits sicker patients but not healthier ones.^4,5,6,7^ More specifically, our TEE score analysis identified patients with 3 or more diseased vessels (vs <3) to be more likely to benefit from TEE, a finding consistent with a 2024 analysis using private administrative claims data.^6^

The survival benefit to TEE among subgroups with greater than 50% left-main disease, 3 or more diseased coronaries, or preoperative inotrope use persisted even among patients with similar PROM scores, suggesting heterogeneity in TEE effectiveness even within lower-risk groups. This finding builds on the study by Metkus et al,^5^ which found no benefit of TEE among patients with low estimated mortality (<4%) by demonstrating that even within similar overall risk categories, certain clinical phenotypes appear more responsive to TEE. While PROM remains a valuable tool for benchmarking surgical outcomes, our findings suggest that PROM scores may be insufficient as the sole basis for identifying patients most likely to benefit from intraoperative TEE. These results underscore the need for more granular, physiology-informed approaches to patient selection—particularly as institutions seek to optimize the use of limited perioperative imaging resources.

Unexpectedly, we found no survival benefit from TEE among patients with CHF. This result contrasts with a 2024 claims-based study showing increased survival in this group with the use of TEE.^6^ This discrepancy may reflect differences in CHF classification using *International Statistical Classification of Diseases and Related Health Problems, Tenth Revision* codes in claims data^6^ compared with New York Heart Association class in this study. Alternatively, this result may indicate that CHF is not actually a modifier of TEE benefit. TEE is particularly valuable for detecting acute, reversible hemodynamic deterioration, such as reduced ejection fraction due to hypotension and decreased coronary perfusion, which may not be as relevant in patients with chronic, fixed ventricular dysfunction due to CHF.^35,36,37^

Another surprising finding from the current study was the absence of TEE benefit at high surgical volume centers. This result contrasts with prior work linking TEE to improved outcomes in higher-risk patients.^4,5,6^ A likely explanation is that high surgical volume hospitals already achieve low mortality (1.72% with TEE vs 1.77% without TEE), potentially offsetting any added value from TEE via factors, such as surgical expertise, intensive care unit resources, and improved staffing. It is also possible that within the high surgical volume centers, institutional practices are already standardized and highly protocolized, thereby diminishing any incremental value of additional intraoperative monitoring with TEE. Although high-volume hospitals vary in size and resources, the absence of any detectable benefit across this stratum suggests that the utility of TEE may plateau once a certain threshold of institutional capability to improve outcomes is reached. In contrast, the more pronounced benefit in the low and medium surgical volume hospitals may reflect greater variability in intraoperative management and outcomes, creating an opportunity for TEE to meaningfully play a role in care decisions.

An alternative explanation may be residual unobserved confounding at the clinician level. One critical unmeasured confounder may be the cardiac anesthesiologist. While not directly measured in this study or previous studies due to the lack of anesthesiologist identifiers,^4,5,6,7^ cardiac anesthesiologists (who often staff high surgical volume centers) may affect postoperative outcomes more than the TEE itself. Specifically, cardiac anesthesiologist involvement may encompass a bundle of co-occurring practices such as goal-directed hemodynamic management, enhanced communication with the surgical team, use of inotropic or vasopressor titration protocols, real-time biventricular function assessments, and proactive optimization of volume status and afterload modification in the context of cardiopulmonary bypass and myocardial protection strategies. Thus, in these settings, TEE may offer only limited incremental benefit because outcomes at high surgical volume centers are already optimized through alternative mechanisms. In contrast, at the low and medium surgical volume centers (where previous research has shown that cardiac anesthesiologist staffing is associated with high likelihood of TEE use during CABG^8^), TEE may serve as a marker for broader cardiac anesthesiologist involvement. Consequently, the observed clinical benefit associated with TEE use at the low surgical volume centers may, in part, reflect the impact of anesthesiologist expertise along with the TEE.

Our findings have practical implications. At lower surgical volume centers, key patient-level factors (eg, >50% left-main coronary stenosis, ≥3 diseased coronaries, and inotrope use) are routinely captured in the electronic medical record. These patient-level parameters could be used as a clinical decision support to guide TEE use or cardiac anesthesiologist staffing, particularly in TEE resource–limited settings. For instance, if a hospital only has the capacity to use intraoperative TEE in 25% of isolated CABGs, targeted TEE allocation to patients with TEE-responsive conditions may improve outcomes. To support this allocation, we developed a clinical software tool that calculates a TEE benefit score based on preoperative characteristics and hospital surgical volume (eAppendix 12 in Supplement 1).

While our clinical decision support tool was developed to guide targeted TEE use in higher-risk patients, our findings also highlight an opportunity to reduce routine TEE use in cases with lower-risk for CABG. Specifically, the findings raise important considerations for adopting a more judicious, risk-stratified approach to intraoperative TEE, particularly among patients with a low likelihood of postoperative complications. Avoiding routine TEE in these populations may offer several advantages. First, from an operational perspective, it may improve operating room efficiency by eliminating the time required for probe placement and image acquisition and interpretation. Second, while life-threatening complications are rare,^38,39^ structural heart literature has reported subclinical or endoscopically evident esophageal injuries in up to 86% of patients,^40^ suggesting a need for caution even in the absence of overt harm. Third, selective TEE use may reduce resource demands, including equipment wear, sterilization time, and staffing, which is particularly relevant for hospitals facing capacity or personnel constraints. Taken together, these considerations support a pragmatic care model in which TEE is not used by default in low-risk isolated CABG but remains readily available for intraoperative hemodynamic changes or unanticipated events. This risk-stratified, on-demand approach aligns with TEE practices in noncardiac surgery and may help optimize the balance between clinical benefit, procedural risk, and resource utilization in cardiac surgical care.

Fourth, the lack of observed survival benefit from intraoperative TEE among patients who underwent isolated CABG at high surgical volume centers (including patients in higher-risk subgroups) suggests a potential opportunity to further explore the comparative effectiveness of TEE in this setting. While the logistics and cost of a large randomized clinical trial would be considerable, our findings highlight a subgroup of patients in whom the marginal benefit of TEE appears limited, making randomized evaluation ethically and clinically justifiable.

### Limitations

This study must be interpreted with awareness of its limitations. First, the observational, nonrandomized design of this study cannot confirm a causal association between TEE and increased survival because of the inability to eliminate residual confounding between patients who underwent surgery with TEE vs without TEE. Given the granularity of patient-level factors captured in the STS ACSD and the rigorous matching techniques used in this study, the most likely source of residual unobserved confounding is clinician level (as opposed to patient level). Examples of clinician-level confounding could be surgeon preference for (or against) intraoperative TEE or the availability (or unavailability) of a cardiac anesthesiologist with the certification to perform an intraoperative TEE. Because the STS ACSD does not include detailed clinician- or hospital-level variables, we stratified hospitals by surgical volume, which is a well-established proxy for institutional capability. We then matched patients within each volume stratum to mitigate confounding related to unmeasured institutional practices.

Second, while we purposefully conducted our analyses within each of the 3 surgical volume categories, we were unable to adjust for a wide variety of hospital-level factors that could confound the association between TEE and increased survival, such as postoperative or intraoperative care process differences between hospitals. Third, the STS ACSD does not include sufficient detail to classify CABG procedures as guideline-indicated or nonindicated with respect to percutaneous coronary intervention candidacy or SYNTAX scoring. However, the matched analyses balanced coronary anatomy across the with TEE and without TEE comparison groups, and we found that patients with left-main or multivessel disease appeared to derive greater benefit from TEE regardless of surgical volume. Fourth, while the STS ACSD does not include a variable for return to cardiopulmonary bypass across all data versions, we conducted a supplementary analysis of unplanned coronary reintervention as a proxy for certain TEE-detected intraoperative findings. Although not a perfect surrogate, this outcome provides insight into potential downstream consequences of TEE use during CABG.

Fifth, we were unable to compare gastroesophageal complication rates between patients who underwent isolated CABG with TEE vs without TEE. While life-threatening complications directly attributable to intraoperative TEE are rare (<0.01%),^38,39^ interventional cardiology literature has reported a gastroesophageal injury rate that is much higher (22% to 40%)^40,41^ than has been previously reported in the cardiac surgical literature (0.1% to 0.4%).^38,39^ Consequently, a key question for future investigation is whether the benefits of intraoperative TEE as an invasive hemodynamic monitor outweigh the risks of gastroesophageal injury in patients who underwent isolated CABG surgery.

## Conclusions

Among patients who underwent isolated CABG at low and medium surgical volume hospitals, the survival benefit of intraoperative TEE was greatest in those with greater than 50% left-main coronary stenosis, 3 or more diseased vessels, or preoperative inotropic support. In contrast, no survival benefit was observed at high surgical volume hospitals, either overall or within high-risk subgroups. These findings support a more individualized approach to TEE use and underscore the need for a randomized clinical trial to define TEE’s role in isolated CABG surgery.

## Notes

Supplement 1. **eAppendix 1.** List of Matching Covariates **eAppendix 2.** Descriptive Analysis **eFigure 1.** Density Plot of the Proportion of CABG Surgeries Using TEE by Hospital Surgical Volume **eTable 1.** Covariate Distribution Among Training and Testing Data, Stratified by Receipt of TEE **eAppendix 3.** Details on Statistical Matching Methodology **eAppendix 4.** Methods for Identification of Subgroups That Benefit From TEE (Stage One) **eAppendix 5.** Methods for Validating TEE Score (Stage Two) **eAppendix 6.** Preliminary Results **eTable 2.** 30-Day Mortality in the Training Data Set Stratified by Hospital Volume and TEE Receipt **eAppendix 7.** Low Surgical Volume Hospitals **eTable 3.** Low Volume Covariate Distribution Among Post-Match Testing Data **eTable 4.** Low Volume Hospitals Training Data Selected Covariate Distribution by TEE Score **eAppendix 8.** Medium Surgical Volume Hospitals **eTable 5.** Medium Volume Covariate Distribution Among Post-Match Testing Data **eTable 6.** Medium Volume Hospitals Training Data Selected Covariate Distribution by TEE Score **eAppendix 9.** High Surgical Volume Hospitals **eTable 7.** High Surgical Volume Hospitals Covariate Distribution Among Post-Match Testing Data **eTable 8.** High Surgical Volume Hospitals Training Data Selected Covariate Distribution by TEE Score **eAppendix 10.** Testing Data Results **eTable 9.** Characteristics Associated With Predicted Individualized TEE Treatment Effects at Low Surgical Volume Hospitals **eTable 10.** Characteristics Associated With Predicted Individualized TEE Treatment Effects at Medium Surgical Volume Hospitals **eAppendix 11.** Additional Outcome Analyses **eAppendix 12.** Risk Score Calculator **eAppendix 13.** Code Documentation
eReferences
**eAppendix 14. **Two-stage Target Trial Matched Analysis

Supplement 2.
Data Sharing Statement
