# Supplementary material for: Surgical Volume and Outcomes of Intraoperative Transesophageal Echocardiography in Coronary Artery Bypass Graft
Source: JAMA Netw Open. 2025 Oct 30;8(10):e2540559. doi: 10.1001/jamanetworkopen.2025.40559 (PMC12576493; doi:10.1001/jamanetworkopen.2025.40559)

Supplemental Online Content

MacKay EJ, Talham CJ, Szeto WY, et al. Surgical volume and outcomes of intraoperative echocardiography outcome in coronary artery bypass graft. *JAMA Netw Open*. 2025;8(10):e2540559. doi:10.1001/jamanetworkopen.2025.40559

eAppendix 1. List of Matching Covariates
eAppendix 2. Descriptive Analysis
eFigure 1. Density Plot of the Proportion of CABG Surgeries Using TEE by Hospital Surgical Volume
eTable 1. Covariate Distribution Among Training and Testing Data, Stratified by Receipt of TEE
eAppendix 3. Details on Statistical Matching Methodology
eAppendix 4. Methods for Identification of Subgroups That Benefit From TEE (Stage One)
eAppendix 5. Methods for Validating TEE Score (Stage Two)
eAppendix 6. Preliminary Results
eTable 2. 30-Day Mortality in the Training Data Set Stratified by Hospital Volume and TEE Receipt
eAppendix 7. Low Surgical Volume Hospitals
eTable 3. Low Volume Covariate Distribution Among Post-Match Testing Data **eTable 4.** Low Volume Hospitals Training Data Selected Covariate Distribution by TEE Score
eAppendix 8. Medium Surgical Volume Hospitals
eTable 5. Medium Volume Covariate Distribution Among Post-Match Testing Data
eTable 6. Medium Volume Hospitals Training Data Selected Covariate Distribution by TEE Score
eAppendix 9. High Surgical Volume Hospitals
eTable 7. High Surgical Volume Hospitals Covariate Distribution Among Post-Match Testing Data
eTable 8. High Surgical Volume Hospitals Training Data Selected Covariate Distribution by TEE Score
eAppendix 10. Testing Data Results
eTable 9. Characteristics Associated With Predicted Individualized TEE Treatment Effects at Low Surgical Volume Hospitals
eTable 10. Characteristics Associated With Predicted Individualized TEE Treatment Effects at Medium Surgical Volume Hospitals

© 2025 MacKay EJ et al. *JAMA Network Open*

eAppendix 11. Additional Outcome Analyses
eAppendix 12. Risk Score Calculator
eAppendix 13. Code Documentation
eReferences
eGraphical abstract. Two-Stage Target Trial Matched Analysis

This supplemental material has been provided by the authors to give readers additional information about their work.

© 2025 MacKay EJ et al. *JAMA Network Open*

1 eAppendix 1. List of matching covariates

Covariate[*†‡]		STS ACSD Long Name[§]	STS ACSD Short Name[‖]
Age (yr)		Patient Age	Age
Gender	Female	Sex	Gender
	Male		
Race	White	Race – Multi-Select	RaceMulti
	Black		
	Asian		
	American Indian / Alaskan		
	Hawaiian / Pacific Islander		
	Other		
BMI		Calculated BMI	CalculatedBMI
Admit Source	Elective	Admit Source	AdmitSrc
	Emergency		
	Transfer		
	Other		
Arrhythmia		Cardiac Arrhythmia	Arrhythmia
Afib. Paroxysmal		Atrial Fibrillation – Type	ArrhythAFib
Afib. Persistent		Atrial Fibrillation – Type	ArrhythAFib
Chronic Lung Disease	None	RF-Chronic Lung Disease	ChrLungD
	Mild		
	Moderate		
	Severe		
	Severity unknown		
CHF by NYHA[¶ #]	None	Classification-NYHA	ClassNYHA
	Class I		
	Class II		
	Class III		
	Class IV		
CVD		Cerebrovascular Disease	CVD
CVA		Cerebrovascular Accident	CVA
Dialysis[¶]		RF-Renal Fail-Dialysis	Dialysis
Liver Disease		RF-Liver Disease	LiverDis
OSA		RF-Sleep Apnea	SlpApn
PASP (mmHg)		Hemo-PA Systolic Pressure	PASYSMeas
PVD		RF-Peripheral Arterial Disease	PVD
Previous PCI		Previous PCI	POCPI
PCI w/in 6 hrs		Previous PCI-Interval	POCPCIIn
PCI stent		Previous PCI-Stent	POCPCISt
PCI indication	No PCI	Previous PCI-Indication For Surgery	POCPCIndSurg
	Complication w/deterioration		
	Complication w/out deterioration		
	Staged for STEMI		

	Staged w/out STEMI		
	Other		
Time Cath to Surgery (days)		Cathtosurg	Cathtosurg
Previous CABG[¶]		Prev CAB	PrCAB
Redo Surgery[¶]		Incidence	Incidenc
Hemoglobin		Hemoglobin	RFHemoglobin
Platelets		Platelet Count	Platelets
Albumin		Total Albumin	TotAlbumin
INR		INR	INR
Creatinine		Last Creatinine Level	CreatLst
Left Main >50% stenosis		Left Main Stenosis >50% Known	StenLeftMain
STEMI / NSTEMI[¶]		Cardiac Presentation/Symptoms – At Time of Admission	CardSympTimeOfAdm
Cardiogenic Shock[¶]		Cardiogenic Shock	CarShock
Number of Diseased Vessels	One	Num Dis Vessels	NumDisV
	Two		
	Three		
ACEI / ARB w/in 48 Hrs.		Meds-ACE Inhibitors or ARB within 48 Hours	MedACEI48
Inotropes w/in 48 Hrs.		Meds-Inotropes Within 48 Hours	MedInotr
Aortic valve insufficiency	None	Aortic Valve Regurgitation Degree	VDnsufA
	Trivial / trace		
	Mild		
	Moderate		
	Severe		
Operative Status	Elective		
	Urgent		
	Emergent		
EF (%)		Hemo Data-EF	HDEF
Predicted Mortality[#, **] (%)		Predicted Risk of Mortality	PredMort

Abbreviations: **STS**: Society of Thoracic Surgeons; **ACSD**: Adult Cardiac Surgery Database; **Yr.** Year; **Afib.**: atrial fibrillation; **CHF**: congestive heart failure; **NYHA**: New York Heart Association Class; **CVD**: Cerebrovascular Disease; **CVA**: Cerebrovascular Accident; **OSA**: obstructive sleep apnea; **PASP**: Pulmonary arterial systolic pressure; **PVD**: Peripheral vascular disease; **PCI**: Percutaneous coronary intervention; **CABG**: coronary artery bypass graft; **STEMI**: ST elevation myocardial infarction; **NSTEMI**: Non-ST segment elevation myocardial infarction; **ACEI**: Angiotensin-converting-enzyme inhibitor; Angiotensin II receptor blocker; **Hrs.** Hours; **EF**: Ejection fraction.

* Covariate label used for matching in all matched analyses

† Covariates matched within strata of each year for the low, medium, and high surgical volume hospitals

‡ For the high surgical volume hospitals, in addition to matching within strata for each year, covariates were matched within quartiles of STS mortality risk

§ Indicates the long name for the STS ACSD source covariate

|| Indicates the short name for the STS ACSD source covariate

¶ Indicates the covariates used for fine balancing among medium and high surgical volume hospitals

# Indicates the covariates also used for fine balancing among low surgical volume hospitals

** Indicates the covariates used for exact match among medium and high surgical volume hospitals

2 eAppendix 2. Descriptive analysis

2.1 TEE use by hospital volume

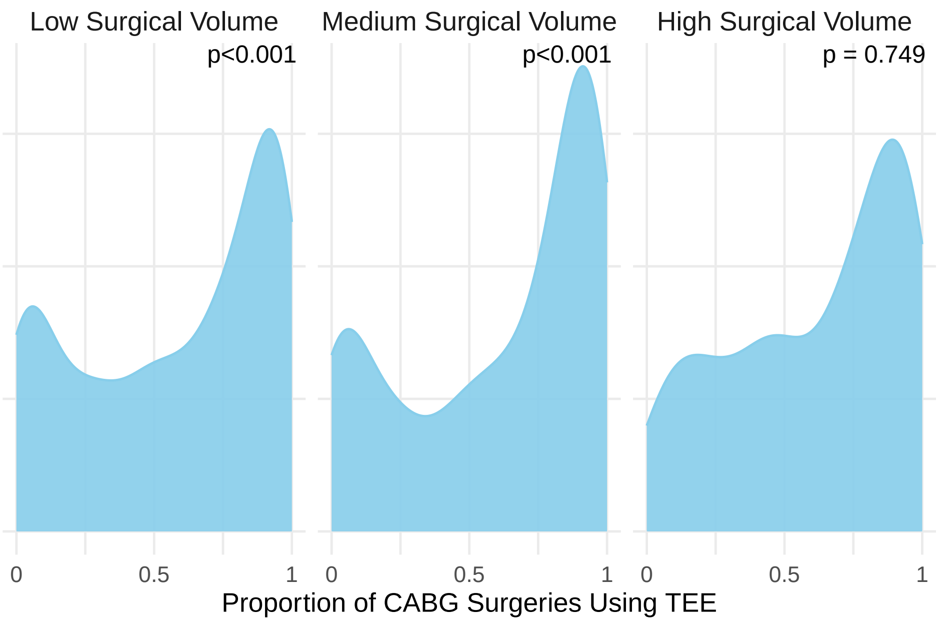

eFigure 1: Density plot of the proportion of CABG surgeries using TEE by hospital surgical volume. P-values are associated with the dip test of unimodality (Hartigan and Hartigan, 1985).

2.2 Covariate distribution of STS data

eTable 1: Covariate distribution among training and testing data, stratified by receipt of TEE. Mean (standard deviation) is presented for continuous variables and count (%) presented for categorical variables.

	**Overall (n = 1,266,055)**	**No TEE (n = 489,895)**	**TEE (n = 776,160)**
Age (yr)	65.66 (10.02)	65.69 (10.05)	65.64 (10.00)
Female	302,079 (23.9)	119,864 (24.5)	182,215 (23.5)
Male	963,976 (76.1)	370,031 (75.5)	593,945 (76.5)
American Indian/Alaskan Native	8,484 (0.7)	3,547 (0.7)	4,937 (0.6)
Asian	45,668 (3.6)	14,140 (2.9)	31,528 (4.1)
Black	94,324 (7.5)	36,395 (7.4)	57,929 (7.5)
White	1,067,823 (84.3)	419,472 (85.6)	648,351 (83.5)
Other	52,312 (4.1)	17,397 (3.6)	34,915 (4.5)
BMI	30.03 (5.91)	30.11 (5.95)	29.98 (5.89)
Admit source			
Elective	591,748 (46.7)	237,428 (48.5)	354,320 (45.7)
Emergency	335,980 (26.5)	131,432 (26.8)	204,548 (26.4)
Transfer	311,583 (24.6)	111,275 (22.7)	200,308 (25.8)
Other	26,744 (2.1)	9,760 (2.0)	16,984 (2.2)
Arrhythmia	185,899 (14.7)	67,462 (13.8)	118,437 (15.3)

eTable 1 continued from previous page

	**Overall (n = 1,266,055)**	**No TEE (n = 489,895)**	**TEE (n = 776,160)**
Afib paroxysmal	97,555 (7.7)	35,098 (7.2)	62,457 (8.0)
Afib persistent	29,743 (2.3)	11,091 (2.3)	18,652 (2.4)
CHF by NYHA	250,277 (19.8)	86,571 (17.7)	163,706 (21.1)
CVD	278,608 (22.0)	104,953 (21.4)	173,655 (22.4)
CVA	100,998 (8.0)	37,570 (7.7)	63,428 (8.2)
Dialysis	40,495 (3.2)	13,769 (2.8)	26,726 (3.4)
Liver	36,634 (2.9)	13,481 (2.8)	23,153 (3.0)
OSA	207,216 (16.4)	78,793 (16.1)	128,423 (16.5)
PA systolic	30.93 (6.79)	30.89 (6.46)	30.95 (6.99)
PVD	171,729 (13.6)	65,334 (13.3)	106,395 (13.7)
Previous PCI	386,030 (30.5)	150,074 (30.6)	235,956 (30.4)
Platelets	220,094 (67,668)	219,798 (67,392)	220,281 (67,841)
Left main >50% stenosis	405,296 (32.0)	154,524 (31.5)	250,772 (32.3)
STEMI/NSTEMI	425,192 (33.6)	157,438 (32.1)	267,754 (34.5)
Num. diseased vessels =3	974,761 (77.0)	371,480 (75.8)	603,281 (77.7)
ACE I w/in 48 hours	475,755 (37.6)	193,751 (39.5)	282,004 (36.3)
Operative status			
Elective	478,614 (37.8)	193,351 (39.5)	285,263 (36.8)
Emergent	46,519 (3.7)	18,094 (3.7)	28,425 (3.7)
Urgent	740,922 (58.5)	278,450 (56.8)	462,472 (59.6)
MIDCAB	18,841 (1.5)	8,993 (1.8)	9,848 (1.3)
OPCAB	149,037 (11.8)	63,138 (12.9)	85,899 (11.1)

3 eAppendix 3. Details on statistical matching methodology

Statistical matching is commonly used to create treated and control groups with similar distributions of covariates. We define some terminology in section 2.1 and provide implementation details in section 2.2.

3.1 Glossary of matching terms

Bipartite Matching: Matching subjects based on a binary treatment status.

Tripartite Matching: Matching control subjects to treated subjects based on a tripartite network. A tripartite network consists of two bipartite networks–a left network and a right network, where the right network is a mirror copy of the left network in nodes, but with possibly different distance structure. Typically the left network is responsible for close pairing and the right network is responsible for balancing (Zhang et al., 2023).

Optimal Matching: Matching control subjects to treated subjects such that some predefined sum of treatment-to-control distances is minimized.

Propensity Score: The propensity score is the conditional probability of assignment to a particular treatment given a vector of observed covariates (Rosenbaum and Rubin, 1983).

Optimal Matching Within Propensity Score Calipers: A hybrid matching method that minimizes the total treatment-to-control distances subject to the constraint that matched cases and control units differ in their estimated propensity scores by no more than a value known as the "caliper" (Rosenbaum and Rubin, 1985). Rosenbaum and Rubin (1985) found that this hybrid method is superior to metric-based matching and propensity score matching.

Mahalanobis Distance: A multivariate measure of covariate distance between units in a sample (Mahalanobis, 1936, Rubin, 1980). The squared Mahalanobis distance is equal to the difference in covariate values of treated units and matched control units, divided by the covariate's standard deviation. Mahalanobis distance takes into account the correlation structure among covariates. The distance is zero if two units have the same value for all covariates and increases as two units become more dissimilar.

Exact Matching: Matching treated subjects to control subjects requiring the same value of a nominal covariate (Rosenbaum, 2002).

Fine Balance: A matching technique that balances exactly the marginal distribution of one nominal variable or the joint distribution of several nominal variables in the treated and control groups after matching (Rosenbaum et al., 2007; Yu et al., 2020).

3.2 Implementation details

We performed a tripartite network-flow based optimal matching procedure that constructs treated and control groups with similar distributions of covariates and simultaneously produces matches that are homogeneous on a few select covariates (Zhang et al., 2023). In the low surgical volume cohort, we matched the training data within strata defined by year of surgery. In the medium and high surgical volume hospital cohorts, we matched the training data within strata defined by year of surgery and

STS predicted mortality quantile. Similarly, for the testing data, we defined strata by year of surgery and TEE score for low surgical volume hospitals and by year of surgery, STS predicted mortality quantile and TEE score for medium and high surgical volume hospitals. We performed matching using the R package `match2C` and the following implementation details:

1. The propensity score was estimated via logistic regression using the R package `stats` on each strata.

2. The left network was responsible for creating matches that are closely paired based on selected covariates (see eAppendix 1 for list of variables). We used the function `create_list_from_scratch` to construct a sparse representation of treated-to-control distances for the left side of the tripartite network using the default, Mahalanobis distance. We set the `X` argument to a matrix including all our covariates. To reduce the computational burden of the match on the training (testing) data, we set the caliper to 0.3 (0.5) with the option `caliper` and set `k` to 300 (500) to restrict potential TEE treated matches to the 300 (500) candidates with the closest propensity score to the untreated (non-TEE) unit.

3. Treatment-to-control distances for the right side of the tripartite network were the sum of two distance calculations. The first was used to balance the distribution of selected covariates between treated and control groups. We used the function `create_list_from_scratch` with the argument `X` set to the propensity score and `method` to 'L1' to calculate treatment-to-control distance based on the L1 norm. To reduce the computational burden of the match on the training (testing) data, we set the caliper to 0.3 (0.5) with the option `caliper` and set `k` to 300 (500).

4. The right network was also responsible for finely balancing the distribution of specified covariates (see eAppendix 1 for list of fine balance variables). To do this, we again used the function `create_list_from_scratch` with the argument `X` set to a matrix of the six fine balance variables and `method` to '0/1' which sets the treatment-to-control distance to 0 if the treated and control units have the same value for the fine balance variables and to 1 otherwise. To reduce the computational burden of the match on the training (testing) data, we set the caliper to 0.3 (0.5) with the option `caliper` and set `k` to 300 (500).

5. We used the function `match_2C_list` to perform 1-to-1 pair matching based on our left and right network treatment-to-control distance lists.

4 eAppendix 4. Methods for identification of subgroups that benefit from TEE (stage one)

4.1 Estimate conditional average treatment effect

We estimate the conditional average treatment effect (CATE) by way of parametric G-estimation. Let $\theta_a = \mathbb{E}\left[Y \mid A = a, W\right]$ where W represents the following covariates,

1. CHF,
2. three diseased vessels,
3. EF < 55,
4. creatinine,
5. $> 50\%$ left main stenosis and
6. inotropes w/in 48 hours of surgery.

We fit logistic regression models $\hat{\theta_0}$ and $\hat{\theta_1}$ for θ_0 and θ_1, respectively, in which we controlled for the six covariates above and an interaction term between CHF and number of diseased vessels. Then, we estimate the CATE for each observation in the training data as,

$$\widehat{C(W_i)} = \hat{\theta}_1(W_i) - \hat{\theta}_0(W_i).$$

4.2 Assign TEE score in training data

We assigned a TEE recommendation score between 1 (most benefit) and 5 (least benefit) based on even quantiles of the CATE distribution.

5 eAppendix 5. Methods for validating TEE score (stage two)

5.1 Assign TEE score in testing data

We predicted the CATE for observations in the testing data set using the model $\widehat{C(W)}$ from step 1. Then, using the quantile cutoffs from step 1, we assigned a TEE recommendation score to each of the observations in the testing data set.

5.2 Validate

To validate the TEE score estimated in stage one, we performed a McNemar test on the matched testing data to compare the outcome between matched pairs for each TEE score group and overall for all scores. We additionally calculated the risk difference and report confidence intervals and p-values for each statistic.

6 eAppendix 6. Preliminary results

6.1 Overall results for training data

eTable 2: 30-day mortality in the training data set stratified by hospital volume and TEE receipt. Risk differences and odds ratios presented.

Hospital Volume	TEE	N	Deaths	Mort.	Risk Diff. (95% CI)	P value	OR (95% CI)	P value
Low	0	20,988	681	3.24%				
Low	1	20,988	593	2.83%	-0.419% (-0.742%, -0.097%)	0.011	0.863 (0.769, 0.968)	0.012
Medium	0	54,329	1,297	2.39%				
Medium	1	54,329	1,169	2.15%	-0.236% (-0.41%, -0.061%)	0.008	0.896 (0.825, 0.973)	0.009
High	0	42,740	755	1.77%				
High	1	42,740	707	1.65%	-0.112% (-0.284%, 0.059%)	0.199	0.933 (0.839, 1.038)	0.208

7 eAppendix 7. Low surgical volume hospitals

7.1 Training data post-match covariate distribution

eTable 3: Low volume covariate distribution among post-match testing data. Mean and standard deviation are reported for continuous variables. Counts and percentages are reported for binary and categorical variables.

	**No TEE (n = 20,988)**	**TEE (n = 20,988)**	**Standardized mean difference**
Age (yr)	66.65 (10.05)	66.75 (9.99)	0.007
Female	5,490 (26.2)	5,414 (25.8)	-0.006
Male	15,498 (73.8)	15,574 (74.2)	
Asian	752 (3.58)	901 (4.29)	-0.024
Black	1,405 (6.69)	1,132 (5.39)	0.037
American Indian/Alaskan Native	154 (0.73)	163 (0.78)	-0.003
White	17,865 (85.1)	17,904 (85.3)	-0.004
Other race	822 (3.92)	889 (4.24)	-0.011
BMI	30.10 (6.11)	30.02 (6.09)	-0.01
Admit source			
Elective	9,678 (46.1)	9,841 (46.9)	0.011
Emergency	7,951 (37.9)	7,643 (36.4)	-0.022
Transfer	2,890 (13.8)	3,008 (14.3)	0.011
Other	469 (2.2)	496 (2.4)	0.006
Arrhythmia	3,208 (15.3)	3,122 (14.9)	-0.008
Afib paroxysmal	1,646 (7.8)	1,595 (7.6)	-0.007
Afib persistent	523 (2.5)	518 (2.5)	-0.001
Chronic lung disease			
None	15,134 (72.1)	14,994 (71.4)	-0.011
Mild	2,182 (10.4)	2,415 (11.5)	0.026
Moderate	1,143 (5.4)	1,202 (5.7)	0.009
Severe	1,016 (4.8)	1,070 (5.1)	0.009
Severity unknown	1,513 (7.2)	1,307 (6.2)	-0.029
CHF by NYHA	3,492 (16.6)	3,397 (16.2)	-0.009
CVD	4,711 (22.4)	4,593 (21.9)	-0.010
CVA	1,702 (8.1)	1,639 (7.8)	-0.008
Dialysis	770 (3.7)	731 (3.5)	-0.007
Liver disease	568 (2.7)	578 (2.8)	0.002
OSA	3,054 (14.6)	2,959 (14.1)	-0.009
PASP (mmHg)	31.17 (6.79)	31.09 (6.87)	-0.008
PVD	3,086 (14.7)	3,042 (14.5)	-0.004
Previous PCI	6,406 (30.5)	6,408 (30.5)	0.0001
PCI w/in 6 hours	257 (1.2)	238 (1.1)	-0.006
PCI stent	5,495 (26.2)	5,501 (26.2)	0.0005
PCI indication			

eTable 3 continued from previous page

	No TEE (n = 20,988)	TEE (n = 20,988)	Standardized mean difference
No PCI	19,988 (95.2)	19,962 (95.1)	-0.008
Complication with deterioration	192 (0.9)	199 (0.9)	0.001
Complication w/out deterioration	260 (1.2)	257 (1.2)	-0.001
Staged w/out STEMI	136 (0.6)	114 (0.5)	-0.009
Staged for STEMI	352 (1.7)	352 (1.7)	9.07e-6
Other reason	60 (0.3)	104 (0.5)	0.012
Time cath to surgery (days)	81.70 (6,467)	18.94 (325)	-0.007
Previous CABG	314 (1.5)	306 (1.5)	-0.002
Redo surgery	372 (1.8)	374 (1.8)	0.001
Hemoglobin	13.13 (2.07)	13.15 (2.00)	0.006
Platelets	219,283.94 (69,816)	218,229.84 (67,811)	-0.011
Albumin	3.71 (0.52)	3.71 (0.52)	0.001
INR	1.06 (0.22)	1.05 (0.20)	-0.006
Creatinine	1.23 (1.16)	1.22 (1.14)	-0.004
Left main >50% stenosis	7,136 (34.0)	7,193 (34.3)	0.004
STEMI/NSTEMI	7,420 (35.4)	7,534 (35.9)	0.008
Cardiogenic shock	449 (2.1)	445 (2.1)	-0.001
Num. diseased vessels =3	16,068 (76.6)	16,156 (77.0)	0.007
ACEI/ARB w/in 48 hours	9,085 (43.3)	8,934 (42.6)	-0.010
Inotropes w/in 48 hours	304 (1.4)	301 (1.4)	-0.001
Aortic valve insufficiency			
None	16,890 (80.5)	16,966 (80.8)	0.006
Trace	1,871 (8.9)	1,832 (8.7)	1.22e-4
Mild	375 (1.8)	342 (1.6)	-0.005
Moderate	12 (0.1)	7 (0.0)	-0.009
Severe	1,840 (8.8)	1,841 (8.8)	-0.008
Operative status			
Elective	8,211 (39.1)	8,129 (38.7)	-0.006
Emergent	1,016 (4.8)	946 (4.5)	-0.012
Urgent	11,761 (56.0)	11,913 (56.8)	0.010
EF (%)	51.92 (12.24)	51.90 (12.17)	-0.001
STS predicted mortality (%)	2.12 (3.33)	2.13 (3.25)	0.004
MIDCAB	338 (1.6)	232 (1.1)	-0.031
OPCAB	2,959 (14.1)	2,864 (13.6)	-0.009

7.2 Training data covariate distribution by TEE score

eTable 4: Low volume hospitals training data selected covariate distribution by TEE score. Mean and standard deviation are reported for continuous variables. Counts and percentages are reported for binary and categorical variables.

	**Overall (n = 41,976)**	**1 (n = 8,395)**	**2 (n = 7,985)**	**3 (n = 8,355)**	**4 (n = 8,845)**	**5 (n = 8,396)**
EF (%)	51.91 (12.21)	44.45 (10.83)	50.66 (11.45)	58.12 (8.22)	57.83 (8.44)	48.16 (14.64)
EF below 55%	18,332 (43.7)	7,209 (85.9)	4,375 (54.8)	1,151 (13.8)	1,362 (15.4)	4,235 (50.4)
Normal EF (55 to 70%)	22,955 (54.7)	1,163 (13.9)	3,506 (43.9)	6,994 (83.7)	7,256 (82.0)	4,036 (48.1)
CHF by NYHA	6,889 (16.4)	338 (4.0)	45 (0.6)	36 (0.4)	103 (1.2)	6,367 (75.8)
Creatinine	1.23 (1.15)	2.11 (2.24)	1.05 (0.37)	1.03 (0.24)	0.90 (0.26)	1.06 (0.60)
Left main >50% stenosis	14,329 (34.1)	4,237 (50.5)	3,283 (41.1)	2,809 (33.6)	1,626 (18.4)	2,374 (28.3)
Num. diseased vessels =3	32,224 (76.8)	8,010 (95.4)	7,622 (95.5)	7,145 (85.5)	4,509 (51.0)	4,938 (58.8)
Inotropes w/in 48 hours	605 (1.4)	430 (5.1)	1 (0.0)	2 (0.0)	6 (0.1)	166 (2.0)
STS predicted mortality (%)	2.12 (3.29)	3.28 (4.70)	1.79 (2.17)	1.45 (1.80)	1.30 (1.67)	2.83 (4.27)

8 eAppendix 8. Medium surgical volume hospitals

8.1 Training data post-match covariate distribution

eTable 5: Medium volume covariate distribution among post-match testing data. Mean and standard deviation are reported for continuous variables. Counts and percentages are reported for binary and categorical variables.

	**No TEE (n = 54,329)**	**TEE (n = 54,329)**	**Standardized mean difference**
Age (yr)	65.64 (9.96)	65.84 (9.87)	0.014
Female	13,302 (24.5)	13,209 (24.3)	-0.002
Male	41,027 (75.5)	41,020 (75.7)	
Asian	1,267 (2.33)	1,636 (3.01)	-0.028
Black	3,739 (6.88)	3,198 (5.89)	0.028
American Indian/Alaskan Native	422 (0.78)	243 (0.45)	0.029
White	47,368 (87.2)	47,509 (87.4)	-0.005
Other race	1,674 (3.08)	1,890 (3.48)	-0.015
BMI	30.27 (5.99)	30.20 (5.85)	-0.008
Admit source			
Elective	26,302 (48.4)	26,475 (48.7)	0.005
Emergency	15,259 (28.1)	14,879 (27.4)	-0.011
Transfer	11,615 (21.4)	12,015 (22.1)	0.013
Other	1,153 (2.1)	960 (1.8)	-0.017
Arrhythmia	7,506 (13.8)	7,370 (13.6)	-0.005
Afib paroxysmal	3,930 (7.2)	3,858 (7.1)	-0.003
Afib persistent	1,224 (2.3)	1,219 (2.2)	-4.32e-4
Chronic lung disease			-0.009
None	40,317 (74.2)	39,948 (73.5)	-0.011
Mild	5,646 (10.4)	6,333 (11.7)	0.028
Moderate	2,602 (4.8)	2,614 (4.8)	0.001
Severe	2,134 (3.9)	2,352 (4.3)	0.014
Severity unknown	3,630 (6.7)	3,082 (5.7)	-0.029
CHF by NYHA	9,524 (17.5)	9,382 (17.3)	-0.005
CVD	11,645 (21.4)	11,490 (21.1)	-0.005
CVA	4,158 (7.7)	4,073 (7.5)	-0.004
Dialysis	1,533 (2.8)	1,348 (2.5)	-0.014
Liver disease	1,532 (2.8)	1,501 (2.8)	-0.002
OSA	8,903 (16.4)	8,602 (15.8)	-0.011
PASP (mmHg)	31.00 (6.56)	30.93 (6.61)	-0.008
PVD	7,158 (13.2)	7,075 (13.0)	-0.003
Previous PCI	16,546 (30.5)	16,221 (29.9)	-0.009
PCI w/in 6 hours	575 (1.1)	500 (0.9)	-0.010
PCI stent	14,451 (26.6)	14,241 (26.2)	-0.006
PCI indication			

eTable 5 continued from previous page

	No TEE (n = 54,329)	TEE (n = 54,329)	Standardized mean difference
No PCI	52,245 (96.2)	52,331 (96.3)	0.006
Complication with deterioration	374 (0.7)	371 (0.7)	-4.95e-4
Complication w/out deterioration	482 (0.9)	462 (0.9)	-0.003
Staged w/out STEMI	253 (0.5)	278 (0.5)	0.005
Staged for STEMI	792 (1.5)	800 (1.5)	0.001
Other reason	183 (0.3)	87 (0.2)	-0.025
Time cath to surgery (days)	94.80 (7,082)	29.73 (2,853)	-0.011
Previous CABG	865 (1.6)	746 (1.4)	-0.013
Redo surgery	1,034 (1.9)	866 (1.6)	-0.016
Hemoglobin	13.30 (1.99)	13.32 (1.92)	0.008
Platelets	219,957.83 (66,419.40)	219,305.12 (65,434)	-0.007
Albumin	3.77 (0.49)	3.77 (0.49)	0.005
INR	1.05 (0.22)	1.05 (0.16)	-0.004
Creatinine	1.18 (1.04)	1.16 (0.99)	-0.010
Left main >50% stenosis	17,246 (31.7)	17,170 (31.6)	-0.002
STEMI/NSTEMI	18,103 (33.3)	18,001 (33.1)	-0.002
Cardiogenic shock	920 (1.7)	751 (1.4)	-0.016
Num. diseased vessels =3	41,469 (76.3)	41,812 (77.0)	0.011
ACEI/ARB w/in 48 hours	21,330 (39.3)	21,224 (39.1)	-0.003
Inotropes w/in 48 hours	607 (1.11)	557 (1.03)	-0.006
Aortic valve insufficiency	5,189 (9.6)	5,045 (9.3)	
None	44,187 (81.3)	43,715 (80.5)	-0.015
Trace	5,189 (9.6)	5,045 (9.3)	-0.006
Mild	4,130 (7.6)	4,792 (8.8)	0.031
Moderate	805 (1.5)	759 (1.4)	-0.005
Severe	18 (0.0)	18 (0.0)	7.56e-16
Operative status			
Elective	21,733 (40.0)	21,736 (40.0)	8.01e-5
Emergent	2,239 (4.1)	2,019 (3.7)	-0.015
Urgent	30,357 (55.9)	30,574 (56.3)	0.006
EF (%)	53.01 (11.68)	53.00 (11.78)	-0.001
STS predicted mortality (%)	1.86 (3.13)	1.82 (2.89)	-0.010
MIDCAB	529 (1.0)	662 (1.2)	-0.002
OPCAB	6,251 (11.5)	6,204 (11.4)	0.017

8.2 Training data covariate distribution by TEE score

eTable 6: Medium volume hospitals training data selected covariate distribution by TEE score. Mean and standard deviation are reported for continuous variables. Counts and percentages are reported for binary and categorical variables.

	**Overall (n = 108,658)**	**1 (n = 21,689)**	**2 (n = 21,763)**	**3 (n = 21,633)**	**4 (n = 21,816)**	**5 (n = 21,757)**
EF (%)	53.01 (11.73)	53.13 (11.54)	55.21 (10.30)	54.08 (10.40)	55.35 (11.37)	47.26 (12.90)
Normal EF (55 to 70%)	63,481 (58.4)	12,578 (58.0)	14,768 (67.9)	12,977 (60.0)	15,874 (72.8)	7,284 (33.5)
EF below %55	43,234 (39.8)	8,736 (40.3)	6,550 (30.1)	8,275 (38.3)	5,404 (24.8)	14,269 (65.6)
CHF by NYHA	18,906 (17.4)	459 (2.1)	1,879 (8.6)	96 (0.4)	8,854 (40.6)	7,618 (35.0)
Creatinine	1.17 (1.02)	1.29 (1.32)	1.38 (0.98)	0.90 (0.12)	0.95 (0.66)	1.32 (1.36)
Left main >50% stenosis	34,416 (31.7)	20,706 (95.5)	4,184 (19.2)	96 (0.4)	7,701 (35.3)	1,729 (7.9)
Num. diseased vessels =3	83,281 (76.6)	21,426 (98.8)	21,725 (99.8)	21,537 (99.6)	12,532 (57.4)	6,061 (27.9)
Inotropes w/in 48 hours	1,164 (1.1)	1,164 (5.4)	0 (0.0)	0 (0.0)	0 (0.0)	0 (0.0)
STS predicted mortality (%)	2.45 (1.12)	2.45 (4.37)	1.86 (2.34)	1.10 (1.33)	1.83 (2.97)	1.95 (3.03)

9 eAppendix 9. High surgical volume hospitals

9.1 Training data post-match covariate distribution

eTable 7: High surgical volume hospitals covariate distribution among post-match testing data. Mean and standard deviation are reported for continuous variables. Counts and percentages are reported for binary and categorical variables.

	**No TEE (n = 42,740)**	**TEE (n = 42,740)**	**Standardized mean difference**
Age (yr)	65.76 (10.04)	65.84 (9.95)	0.004
Female	10,413 (24.4)	10,157 (23.8)	-0.010
Male	32,327 (75.6)	32,583 (76.2)	
Asian	1,461 (3.42)	1,492 (3.49)	-0.002
Black	3,564 (8.34)	3,239 (7.58)	0.019
American Indian/Alaskan Native	263 (0.62)	268 (0.63)	-0.001
White	36,064 (84.4)	36,179 (84.6)	-0.005
Other race	1,572 (3.68)	1,721 (4.01)	-0.012
BMI	29.94 (5.88)	29.90 (5.79)	-0.004
Admit source			
Elective	20,842 (48.8)	20,961 (49.0)	0.003
Emergency	8,268 (19.3)	7,696 (18.0)	-0.024
Transfer	12,933 (30.3)	13,268 (31.0)	0.012
Other	697 (1.6)	815 (1.9)	0.014
Arrhythmia	5,901 (13.8)	5,928 (13.9)	0.001
Afib paroxysmal	3,139 (7.3)	3,180 (7.4)	0.002
Afib persistent	1,004 (2.3)	997 (2.3)	-0.001
Chronic lung disease			
None	31,865 (74.6)	32,171 (75.3)	0.012
Mild	4,851 (11.4)	4,738 (11.1)	-0.006
Moderate	1,972 (4.6)	1,811 (4.2)	-0.013
Severe	1,785 (4.2)	1,623 (3.8)	-0.014
Severity unknown	2,267 (5.3)	2,397 (5.6)	0.010
CHF by NYHA	8,371 (19.6)	8,312 (19.4)	-0.002
CVD	9,483 (22.2)	9,410 (22.0)	-0.003
CVA	3,384 (7.9)	3,369 (7.9)	-0.001
Dialysis	1,202 (2.8)	1,078 (2.5)	-0.012
Liver disease	1,251 (2.9)	1,215 (2.8)	-0.004
OSA	7,046 (16.5)	6,950 (16.3)	-0.004
PASP (mmHg)	30.74 (6.28)	30.69 (6.30)	-0.006
PVD	5,951 (13.9)	5,925 (13.9)	-0.002
Previous PCI	13,240 (31.0)	13,090 (30.6)	-0.005
PCI w/in 6 hours	344 (0.8)	311 (0.7)	-0.006
PCI stent	11,802 (27.6)	11,654 (27.3)	-0.005
PCI indication			

eTable 7 continued from previous page

	No TEE (n = 42,740)	TEE (n = 42,740)	Standardized mean difference
No PCI	41,296 (96.6)	41,360 (96.8)	0.011
Complication with deterioration	260 (0.6)	236 (0.6)	-0.002
Complication w/out deterioration	376 (0.9)	368 (0.9)	-0.002
Staged w/out STEMI	184 (0.4)	195 (0.5)	0.002
Staged for STEMI	562 (1.3)	527 (1.2)	-0.016
Other reason	62 (0.1)	54 (0.1)	-0.001
Time cath to surgery (days)	52.75 (4596)	18.73 (298.48)	-0.005
Previous CABG	793 (1.9)	723 (1.7)	-0.008
Redo surgery	924 (2.2)	822 (1.9)	-0.011
Hemoglobin	13.27 (1.94)	13.28 (1.91)	0.004
Platelets	219,680.76 (67,632)	218,981.57 (65,485)	-0.007
Albumin	3.79 (0.50)	3.80 (0.49)	0.010
INR	1.05 (0.25)	1.05 (0.17)	-0.006
Creatinine	1.19 (1.06)	1.17 (0.99)	-0.013
Left main >50% stenosis	13,022 (30.5)	13,142 (30.7)	0.004
STEMI/NSTEMI	0.30 (0.46)	0.30 (0.46)	0.001
Cardiogenic shock	12,886 (30.1)	12,931 (30.3)	-0.016
Num. diseased vessels =3	32,227 (75.4)	32,452 (75.9)	0.002
ACEI/ARB w/in 48 hours	16,076 (37.6)	15,738 (36.8)	-0.012
Inotropes w/in 48 hours	373 (0.9)	373 (0.9)	1.67e-4
Aortic valve insufficiency			
None	34,148 (79.9)	33,604 (78.6)	-0.021
Trace	4,443 (10.4)	4,627 (10.8)	0.009
Mild	3,517 (8.2)	3,875 (9.1)	0.020
Moderate	615 (1.4)	619 (1.4)	2.73e-4
Severe	17 (0.0)	15 (0.0)	-0.002
Operative status			
Elective	16,184 (37.9)	15,998 (37.4)	-0.006
Emergent	1,276 (3.0)	1,401 (3.3)	0.012
Urgent	25,280 (59.1)	25,341 (59.3)	0.002
EF (%)	53.22 (11.31)	53.01 (11.68)	-0.012
STS predicted mortality (%)	1.83 (2.88)	1.86 (3.02)	-0.007
MIDCAB	1,314 (3.1)	620 (1.5)	-0.078
OPCAB	6,129 (14.3)	4,419 (10.3)	-0.087

9.2 Training data covariate distribution by TEE score

eTable 8: High surgical volume hospitals training data selected covariate distribution by TEE score. Mean and standard deviation are reported for continuous variables. Counts and percentages are reported for binary and categorical variables.

	**Overall (n = 85,480)**	**1 (n = 17,067)**	**2 (n = 17,114)**	**3 (n = 16,947)**	**4 (n = 17,082)**	**5 (n = 17,270)**
EF (%)	53.12 (11.50)	45.58 (13.68)	56.88 (9.11)	50.94 (10.71)	57.85 (8.10)	54.29 (10.56)
EF $< 55\%$	33,367 (39.0)	11,575 (67.8)	3,590 (21.0)	9,747 (57.5)	2,292 (13.4))	6,163 (35.7)
CHF by NYHA	16,684 (19.5)	14,483 (84.9)	2,123 (12.4)	4 (0.02)	17 (0.10)	57 (0.33)
Creatinine	1.18 (1.03)	1.32 (1.19)	1.03 (0.63)	0.86 (0.24)	1.23 (0.49)	1.45 (1.71)
Left main $>50\%$ stenosis	26,164 (30.6)	4,596 (26.9)	4,842 (28.3)	12 (0.1)	257 (1.5)	16,457 (95.3)
Num. diseased vessels =3	64,679 (75.7)	13,412 (78.6)	187 (1.1)	16,927 (99.9)	17,019 (99.6)	17,134 (99.2)
Inotropes w/in 48 hours	746 (0.9)	746 (4.4)	0 (0.0)	0 (0.0)	0 (0.0)	0 (0.0)
STS predicted mortality (%)	1.84 (2.95)	3.10 (4.85)	1.28 (1.95)	1.25 (1.53)	1.50 (1.89)	2.07 (2.80)

10 eAppendix 10. Testing data results

10.1 Low surgical volume hospitals

eTable 9: Characteristics associated with predicted individualized TEE treatment effects at low surgical volume hospitals. Mean and standard deviation are reported for continuous variables. Counts and percentages are reported for binary, categorical, and ordinal variables.

	**Overall**		**TEE Score 1**		**TEE Score 2**	
	**no TEE**	**TEE**	**no TEE**	**TEE**	**no TEE**	**TEE**
n	74,320	74,320	13,695	13,695	14,217	14,217
EF	52.54 (11.82)	52.50 (11.91)	44.73 (10.69)	44.65 (10.62)	50.86 (11.10)	50.88 (11.26)
EF <55%	30,732 (41.4)	30,879 (41.5)	11,770 (85.9)	11,886 (86.8)	7,861 (55.3)	7,851 (55.2)
CHF	11,312 (15.2)	11,211 (15.1)	521 (3.8)	487 (3.6)	62 (0.4)	45 (0.3)
Creatinine	1.19 (1.09)	1.18 (1.05)	2.07 (2.23)	2.02 (2.15)	1.04 (0.34)	1.04 (0.33)
>50% left main stenosis	24,574 (33.1)	24,611 (33.1)	6,912 (50.5)	6,961 (50.8)	5,762 (40.5)	5,762 (40.5)
$\geq$ 3 diseased coronaries	56,751 (76.4)	57,135 (76.9)	13,088 (95.6)	13,090 (95.6)	13,652 (96.0)	13,687 (96.3)
Inotropes w/in 48 hours of surgery	925 (1.2)	893 (1.2)	671 (4.9)	650 (4.7)	1 (0.0)	1 (0.0)
STS Predicted Mortality Risk	1.85 (2.91)	1.84 (2.88)	3.00 (4.19)	2.95 (4.06)	1.60 (2.02)	1.56 (1.79)
Mean Death	2.94%	2.47%	5.05%	4.34%	2.75%	2.16%
Risk Difference	-0.47% (-0.631, -0.308)		-0.701% (-1.191, -0.211)		-0.591% (-0.945, -0.236)	
Odds Ratio	0.83 (0.778, 0.885)		0.849 (0.755, 0.953)		0.775 (0.662, 0.938)	
E-value	1.51		1.28		1.33	
	**TEE Score 3**		**TEE Score 4**		**TEE Score 5**	
	**no TEE**	**TEE**	**no TEE**	**TEE**	**no TEE**	**TEE**
n	15,541	15,541	16,477	16,477	14,390	14,390
EF	58.29 (7.95)	58.34 (7.94)	58.00 (8.06)	58.01 (8.08)	49.17 (14.39)	48.97 (14.53)
EF <55%	2,004 (12.9)	1,935 (12.5)	2,360 (14.3)	2,361 (14.3)	6,737 (46.8)	6,846 (47.6)
CHF	67 (0.4)	52 (0.3)	143 (0.9)	119 (0.7)	10,519 (73.1)	10,508 (73.0)
Creatinine	1.03 (0.25)	1.02 (0.23)	0.90 (0.25)	0.89 (0.26)	1.02 (0.52)	1.02 (0.53)
>50% left main stenosis	5,191 (33.4)	5,162 (33.2)	2,891 (17.5)	2,871 (17.4)	3,818 (26.5)	3,855 (26.8)
$\geq$ 3 diseased coronaries	13,387 (86.1)	13,490 (86.8)	8,432 (51.2)	8,609 (52.2)	8,192 (56.9)	8,259 (57.4)
Inotropes w/in 48 hours of surgery	4 (0.0)	3 (0.0)	8 (0.0)	2 (0.0)	241 (1.7)	237 (1.6)
STS Predicted Mortality Risk	1.29 (1.54)	1.30 (1.529)	1.14 (1.51)	1.14 (1.58)	2.42 (3.92)	2.44 (3.99)
Mean Death	2.05%	1.63%	1.68%	1.35%	3.52%	3.18%
Risk Difference	-0.418% (-0.715, -0.121)		-0.334% (-0.595, -0.072)		-0.314% (-0.745, 0.064)	
Odds Ratio	0.79 (0.666, 0.938)		0.796 (0.662, 0.938)		0.895 (0.782, 1.023)	
E-value	1.33		1.33		1	

Abbreviations: TEE: transesophageal echocardiography; EF: ejection fraction; CHF: congestive heart failure; STS: Society of Thoracic Surgeons

10.2 Medium surgical volume hospitals

eTable 10: Characteristics associated with predicted individualized TEE treatment effects at medium surgical volume hospitals. Mean and standard deviation are reported for continuous variables. Counts and percentages are reported for binary, categorical, and ordinal variables.

	**Overall**		**TEE Score 1**		**TEE Score 2**	
	**no TEE**	**TEE**	**no TEE**	**TEE**	**no TEE**	**TEE**
n	163,721	163,721	32,633	32,633	32,842	32,842
EF	53.00 (11.68)	52.99 (11.73)	53.15 (11.35)	53.17 (11.42)	55.04 (10.43)	55.04 (10.36)
EF <55%	64,822 (39.6)	65,426 (40.0)	13,071 (40.1)	13,319 (40.8)	9,893 (30.1)	10,069 (30.7)
CHF	28,540 (17.4)	28,324 (17.3)	696 (2.1)	657 (2.0)	2,981 (9.1)	2,917 (8.9)
Creatinine	1.17 (1.02)	1.16 (0.97)	1.30 (1.32)	1.28 (1.29)	1.39 (0.99)	1.37 (0.95)
>50% left main stenosis	51,914 (31.7)	51,737 (31.6)	31,149 (95.5)	31,289 (95.9)	6,343 (19.3)	6,221 (18.9)
$\geq$ 3 diseased coronaries	124,996 (76.3)	125,551 (76.7)	32,215 (98.7)	32,322 (99.0)	32,788 (99.8)	32,809 (99.9)
Inotropes w/in 48 hours of surgery	1,804 (1.1)	1,645 (1.0)	1,804 (5.5)	1,645 (5.0)	0 (0.0)	0 (0.0)
STS Predicted Mortality Risk	1.83 (3.02)	1.83 (2.98)	2.42 (4.32)	2.41 (4.31)	1.88 (2.48)	1.84 (2.28)
Mean Death	2.34%	2.09%	3.35%	2.88%	2.45%	2.15%
Risk Difference	-0.245% (-0.344, -0.146)		-0.472% (-0.732, -0.212)		-0.298% (-0.525, -0.072)	
Odds Ratio	0.889 (0.847, 0.932)		0.848 (0.774, 0.93)		0.872 (0.785, 0.969)	
E-value	1.35		1.36		1.21	
	**TEE Score 3**		**TEE Score 4**		**TEE Score 5**	
	**no TEE**	**TEE**	**no TEE**	**TEE**	**no TEE**	**TEE**
n	32,099	32,099	33,287	33,287	32,860	32,860
EF	53.97 (10.41)	53.97 (10.33)	55.48 (11.33)	55.43 (11.49)	47.34 (12.82)	47.35 (12.96)
EF <55%	12,414 (38.7)	12,556 (39.1)	8,022 (24.1)	8,057 (24.2)	21,422 (65.2)	21,425 (65.2)
CHF	136 (0.4)	99 (0.3)	13,308 (40.0)	13,299 (40.0)	11,419 (34.8)	11,352 (34.5)
Creatinine	0.90 (0.09)	0.90 (0.09)	0.94 (0.66)	0.94 (0.64)	1.32 (1.34)	1.29 (1.24)
>50% left main stenosis	136 (0.4)	99 (0.3)	11,755 (35.3)	11,742 (35.3)	2,531 (7.7)	2,386 (7.3)
$\geq$ 3 diseased coronaries	31,963 (99.6)	32,000 (99.7)	18,944 (56.9)	19,244 (57.8)	9,086 (27.7)	9,176 (27.9)
Inotropes w/in 48 hours of surgery	0 (0.0)	0 (0.0)	0 (0.0)	0 (0.0)	0 (0.0)	0 (0.0)
STS Predicted Mortality Risk	1.10 (1.31)	1.09 (1.30)	1.80 (2.96)	1.82 (2.95)	1.96 (3.04)	1.95 (3.02)
Mean Death	1.37%	1.17%	2.13%	2.01%	2.37%	2.24%
Risk Difference	-0.206% (-0.377, -0.034)		-0.117% (-0.329, 0.095)		-0.134% (-0.358, 0.091)	
Odds Ratio	0.846 (0.733, 0.975)		0.941 (0.843, 1.052)		0.94 (0.845, 1.044)	
E-value	1.19		1		1	

Abbreviations:TEE: transesophageal echocardiography;EF:ejection fraction;CHF:congestive heart failure:STS:Society of Thoracic Surgeons

11 eAppendix 11. Additional outcome analyses

11.1 Postoperative new-onset atrial fibrillation

Low-Volume								
**TEE Score**	**TEE**	**N**	**Count**	**Mean**	**RD (95% CI)**	**P-value**	**OR (95% CI)**	**P-value**
Pooled	0	74320	17877	24.05%				
Pooled	1	74320	18380	24.73%	0.677% (0.249, 1.105)	0.002	1.039 (1.014, 1.064)	0.002
1	0	13695	3520	25.70%				
1	1	13695	3730	27.24%	1.533% (0.505, 2.562)	0.003	1.085 (1.027, 1.146)	0.004
2	0	14217	3429	24.12%				
2	1	14217	3541	24.91%	0.788% (-0.193, 1.769)	0.115	1.045 (0.989, 1.105)	0.119
3	0	15541	3764	24.22%				
3	1	15541	3891	25.04%	0.817% (-0.119, 1.754)	0.087	1.047 (0.993, 1.104)	0.090
4	0	16477	3617	21.95%				
4	1	16477	3728	22.63%	0.674% (-0.208, 1.555)	0.134	1.041 (0.987, 1.098)	0.138
5	0	14390	3547	24.65%				
5	1	14390	3490	24.25%	-0.396% (-1.368, 0.576)	0.424	0.978 (0.925, 1.033)	0.432

Medium-Volume								
**TEE Score**	**TEE**	**N**	**Count**	**Mean**	**RD (95% CI)**	**P-value**	**OR (95% CI)**	**P-value**
Pooled	0	163721	40937	25.00%				
Pooled	1	163721	42341	25.86%	0.858% (0.565, 1.15)	<0.001	1.048 (1.032, 1.065)	<0.001
1	0	32633	8981	27.52%				
1	1	32633	9198	28.19%	0.665% (-0.012, 1.342)	0.054	1.035 (0.999, 1.072)	0.055
2	0	32842	8507	25.90%				
2	1	32842	8876	27.03%	1.124% (0.461, 1.786)	0.001	1.062 (1.025, 1.100)	0.001
3	0	32099	7569	23.58%				
3	1	32099	7802	24.31%	0.726% (0.081, 1.37)	0.027	1.043 (1.005, 1.082)	0.028
4	0	33287	7890	23.70%				
4	1	33287	8265	24.83%	1.127% (0.492, 1.761)	0.001	1.067 (1.028, 1.106)	0.001
5	0	32860	7990	24.32%				
5	1	32860	8200	24.95%	0.639% (-0.008, 1.286)	0.053	1.036 (0.999, 1.075)	0.054

High-Volume								
**TEE Score**	**TEE**	**N**	**Count**	**Mean**	**RD (95% CI)**	**P-value**	**OR (95% CI)**	**P-value**
Pooled	0	128215	33026	25.76%				
Pooled	1	128215	34404	26.83%	1.075% (0.741, 1.409)	<0.001	1.059 (1.041, 1.079)	<0.001
1	0	25296	6850	27.08%				
1	1	25296	7352	29.06%	1.985% (1.215, 2.754)	<0.001	1.107 (1.064, 1.152)	<0.001
2	0	26372	6385	24.21%				
2	1	26372	6498	24.64%	0.428% (-0.287, 1.144)	0.241	1.025 (0.984, 1.067)	0.245
3	0	25049	5955	23.77%				
3	1	25049	6089	24.31%	0.535% (-0.193, 1.263)	0.15	1.031 (0.989, 1.076)	0.153
4	0	25473	6602	25.92%				
4	1	25473	7012	27.53%	1.61% (0.854, 2.365)	<0.001	1.089 (1.046, 1.133)	<0.001
5	0	26025	7234	27.80%				
5	1	26025	7453	28.64%	0.841% (0.077, 1.606)	0.031	1.043 (1.004, 1.085)	0.032

11.2 Secondary outcomes

11.2.1 Coronary reintervention

	TEE	N	Count	Mean	RD (95% CI)	P-value	OR (95% CI)	P-value
**Low-Volume**								
Pooled	0	74320	267	0.36%				
Pooled	1	74320	302	0.41%	0.047% (-0.016, 0.110)	0.142	1.131 (0.956, 1.339)	0.154
**Medium-Volume**								
	**TEE**	**N**	**Count**	**Mean**	**RD (95% CI)**	**P-value**	**OR (95% CI)**	**P-value**
Pooled	0	163721	639	0.39%				
Pooled	1	163721	623	0.38%	-0.010% (-0.052, 0.033)	0.652	0.975 (0.871, 1.091)	0.672
**High-Volume**								
	**TEE**	**N**	**Count**	**Mean**	**RD (95% CI)**	**P-value**	**OR (95% CI)**	**P-value**
Pooled	0	128215	381	0.30%				
Pooled	1	128215	426	0.33%	0.035% (-0.008, 0.078)	0.112	1.119 (0.972, 1.288)	0.120

11.2.2 New postoperative stroke

	TEE	N	Count	Mean	RD (95% CI)	P-value	OR (95% CI)	P-value
**Low-Volume**								
Pooled	0	74320	1025	1.38%				
Pooled	1	74320	1025	1.38%	0% (-0.118, 0.118)	0.999	1% (0.915, 1.093)	0.999
**Medium-Volume**								
	**TEE**	**N**	**Count**	**Mean**	**RD (95% CI)**	**P-value**	**OR (95% CI)**	**P-value**
Pooled	0	163721	2035	1.24%				
Pooled	1	163721	2081	1.27%	0.028% (-0.048, 0.104)	0.470	1.023 (0.961, 1.089)	0.480
**High-Volume**								
	**TEE**	**N**	**Count**	**Mean**	**RD (95% CI)**	**P-value**	**OR (95% CI)**	**P-value**
Pooled	0	128215	1589	1.24%				
Pooled	1	128215	1595	1.24%	0.005% (-0.081, 0.09)	0.915	1.004 (0.935, 1.077)	0.929

11.2.3 Chest exploration

	TEE	N	Count	Mean	RD (95% CI)	P-value	OR (95% CI)	P-value
**Low-Volume**								
Pooled	0	74320	1387	1.87%				
Pooled	1	74320	1382	1.86%	-0.007 (-0.144, 0.130)	0.923	0.996 (0.923, 1.075)	0.939
**Medium-Volume**								
	**TEE**	**N**	**Count**	**Mean**	**RD (95% CI)**	**P-value**	**OR (95% CI)**	**P-value**
Pooled	0	163721	2771	1.69%				
Pooled	1	163721	2632	1.61%	-0.085% (-0.172, 0.002)	0.056	0.949 (0.899, 1.002)	0.058
**High-Volume**								
	**TEE**	**N**	**Count**	**Mean**	**RD (95% CI)**	**P-value**	**OR (95% CI)**	**P-value**
Pooled	0	128215	2055	1.60%				
Pooled	1	128215	2127	1.66%	0.056% (-0.042, 0.154)	0.260	1.036 (0.974, 1.102)	0.267

12 eAppendix 12. Risk score calculator

The risk score calculator can be found at https://bzhang3.shinyapps.io/TEERecScore/.

13 eAppendix 13. Code documentation

All analyses including data preprocessing, statistical matching, and outcome analyses can be reproduced using the R and Stata code stored in the following GitHub code repository: https://github.com/emily-jane-mackay/STS_TEE_Score.

References

Hartigan, J. A. and Hartigan, P. M. (1985). The dip test of unimodality. *The annals of Statistics*, pages 70–84.

Mahalanobis, P. C. (1936). On the generalized distance in statistics. National Institute of Science of India.

Rosenbaum, P. R. (2002). *Observational Studies.* Springer.

Rosenbaum, P. R., Ross, R. N., and Silber, J. H. (2007). Minimum distance matched sampling with fine balance in an observational study of treatment for ovarian cancer. *Journal of the American Statistical Association*, 102(477):75–83.

Rosenbaum, P. R. and Rubin, D. B. (1983). The central role of the propensity score in observational studies for causal effects. *Biometrika*, 70(1):41–55.

Rosenbaum, P. R. and Rubin, D. B. (1985). Constructing a control group using multivariate matched sampling methods that incorporate the propensity score. *The American Statistician*, 39(1):33–38.

Rubin, D. B. (1980). Bias reduction using mahalanobis-metric matching. *Biometrics*, pages 293–298.

Yu, R., Silber, J. H., Rosenbaum, P. R., et al. (2020). Matching methods for observational studies derived from large administrative databases. *Statistical Science*, 35(3):338–355.

Zhang, B., Small, D. S., Lasater, K. B., McHugh, M., Silber, J. H., and Rosenbaum, P. R. (2023). Matching one sample according to two criteria. *Journal of the American Statistical Association*, 118(542):1140–1151.

14 eAppendix 14: Two-Stage Target Trial Matched Analysis

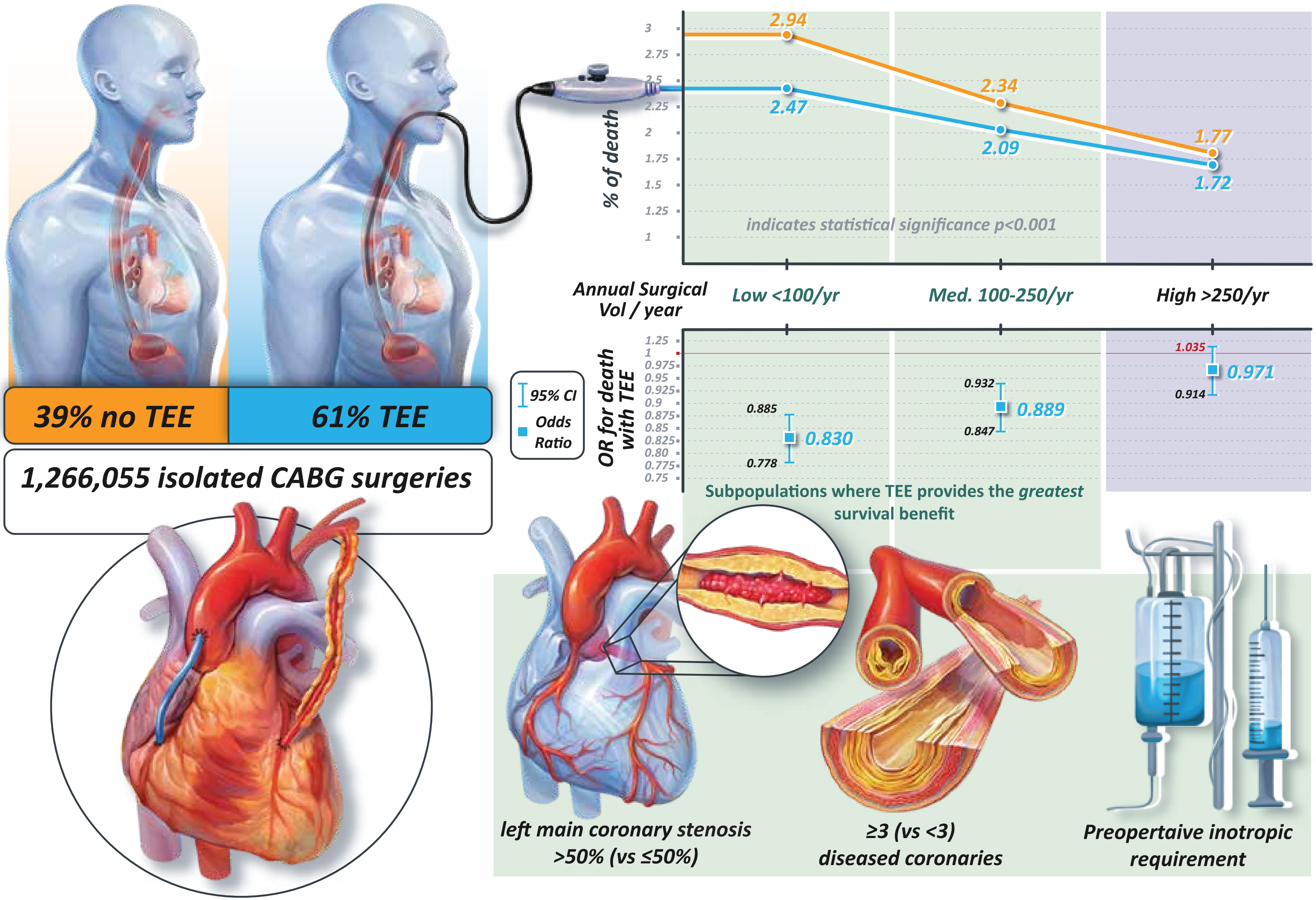

Supplement: Supplement 1. — eAppendix 1. List of Matching Covariates eAppendix 2. Descriptive Analysis eFigure 1. Density Plot of the Proportion of CABG Surgeries Using TEE by Hospital Surgical Volume eTable 1. Covariate Distribution Among Training and Testing Data, Stratified by Receipt of TEE eAppendix 3. Details on Statistical Matching Methodology eAppendix 4. Methods for Identification of Subgroups That Benefit From TEE (Stage One) eAppendix 5. Methods for Validating TEE Score (Stage Two) eAppendix 6. Preliminary Results eTable 2. 30-Day Mortality in the Training Data Set Stratified by Hospital Volume and TEE Receipt eAppendix 7. Low Surgical Volume Hospitals eTable 3. Low Volume Covariate Distribution Among Post-Match Testing Data eTable 4. Low Volume Hospitals Training Data Selected Covariate Distribution by TEE Score eAppendix 8. Medium Surgical Volume Hospitals eTable 5. Medium Volume Covariate Distribution Among Post-Match Testing Data eTable 6. Medium Volume Hospitals Training Data Selected Covariate Distribution by TEE Score eAppendix 9. High Surgical Volume Hospitals eTable 7. High Surgical Volume Hospitals Covariate Distribution Among Post-Match Testing Data eTable 8. High Surgical Volume Hospitals Training Data Selected Covariate Distribution by TEE Score eAppendix 10. Testing Data Results eTable 9. Characteristics Associated With Predicted Individualized TEE Treatment Effects at Low Surgical Volume Hospitals eTable 10. Characteristics Associated With Predicted Individualized TEE Treatment Effects at Medium Surgical Volume Hospitals eAppendix 11. Additional Outcome Analyses eAppendix 12. Risk Score Calculator eAppendix 13. Code Documentation eReferences eAppendix 14. Two-stage Target Trial Matched Analysis [file jamanetwopen-e2540559-s001.pdf]
