# Supplementary material for: Surgical Volume and Outcomes of Intraoperative Transesophageal Echocardiography in Coronary Artery Bypass Graft
Source: JAMA Netw Open. 2025 Oct 30;8(10):e2540559. doi: 10.1001/jamanetworkopen.2025.40559 (PMC12576493; doi:10.1001/jamanetworkopen.2025.40559)
Supplement: Supplement 2. — Data Sharing Statement [file jamanetwopen-e2540559-s002.pdf]

Data Sharing Statement

MacKay. Surgical Volume and Outcomes of Intraoperative Transesophageal Echocardiography in Coronary Artery Bypass Graft. *JAMA Netw Open*. Published October 30, 2025. doi:10.1001/jamanetworkopen.2025.40559

Data
Data available: No

Additional Information
Explanation for why data not available: The data is owned and managed by the Society of Thoracic Surgeons (STS) through the Participant User File (PUF) program
